# A systematic review of neuropsychiatric and cognitive assessments used in clinical trials for amyotrophic lateral sclerosis

**DOI:** 10.1007/s00415-020-10203-z

**Published:** 2020-09-10

**Authors:** Emily Beswick, Emily Park, Charis Wong, Arpan R. Mehta, Rachel Dakin, Siddharthan Chandran, Judith Newton, Alan Carson, Sharon Abrahams, Suvankar Pal

**Affiliations:** 1 Centre for Clinical Brain Sciences, The University of Edinburgh, Edinburgh, Scotland; 2 Anne Rowling Regenerative Neurology Clinic, 49 Little France Crescent, EH16 4SB, Edinburgh, UK; 3 The School of Medicine and Veterinary Medicine, The University of Edinburgh, Edinburgh, Scotland; 4 Euan MacDonald Centre for MND Research, The University of Edinburgh, Edinburgh, Scotland; 5 Human Cognitive Neurosciences, Psychology, School of Philosophy, Psychology and Language Sciences, The University of Edinburgh, Edinburgh, Scotland; 6 UK Dementia Research Institute, The University of Edinburgh, Edinburgh, Scotland

**Keywords:** Amyotrophic lateral sclerosis, Motor neuron disease, Neuropsychiatric, Cognition, Clinical trials

## Abstract

**Background:**

Up to 50% of people with amyotrophic lateral sclerosis (ALS) experience cognitive dysfunction, whilst depression and anxiety are reported in up to 44% and 33%, respectively. These symptoms impact on quality of life, and are associated with a poorer prognosis. Historically, outcomes in clinical trials have focused on the effect of candidate drugs on physical functioning.

**Methods:**

We reviewed the past 25 years of clinical trials of investigative medicinal products in people with ALS, since the licensing of riluzole, and extracted data on frequency and type of assessment for neuropsychiatric symptoms and cognitive impairment. Trial registry databases, including WHO International Trials Registry, European Clinical Trials Register, clinicaltrials.gov, and PubMed, were systematically searched for Phase II, III or IV trials registered, completed or published between 01/01/1994 and 31/10/2019. No language restrictions were applied. Outcome measures, exclusion criteria and assessment tool used were extracted.

**Results:**

216 trials, investigating 26,326 people with ALS, were reviewed. 35% assessed neuropsychiatric symptoms, and 22% assessed cognition, as Exclusion Criteria or Outcome Measures. 3% (*n* = 6) of trials assessed neuropsychiatric symptoms as a Secondary Outcome Measure, and 4% (*n* = 8) assessed cognition as Outcome Measures; only one trial included assessments for both cognition and neuropsychiatric symptoms as Outcome Measures. Three ALS-specific assessments were used in six trials.

**Conclusions:**

Trials for people with ALS have neglected the importance of neuropsychiatric symptoms and cognitive impairment. Evaluation of these extra-motor features is essential to understanding the impact of candidate drugs on all symptoms of ALS.

**PROPSERO registration:**

CRD42020175612.

**Electronic supplementary material:**

The online version of this article (10.1007/s00415-020-10203-z) contains supplementary material, which is available to authorized users.

## Introduction

Amyotrophic lateral sclerosis (ALS) has been traditionally characterised as a disease of the motor system [1]; however, extra-motor symptoms, including neuropsychiatric and cognitive symptoms, are now becoming more widely acknowledged as prevalent [2] and debilitating [3] features of this condition, and are predictive of disability [4].

Findings from a recent population-based study in Scotland reported a prevalence of neuropsychiatric disorders of 19.7% in people with ALS, 70% of which were mood disorders and 31.67% neurotic disorders (inclusive of anxiety, stress-related and somatoform disorders) [5].

The prevalence of these disorders in people with ALS is higher than that in the general population, 6.9% of whom meet thresholds for a depressive disorder and 14% an anxiety disorder [6, 7]. These differences remain when focusing on a group more representative of the general individual with ALS, the older adult population [8].

Cognitive impairment is an additional extra-motor feature of ALS that is highly prevalent, with some form of cognitive or behavioural symptoms experienced by 30–50% of people with ALS [9]. 15% of people with ALS meet diagnostic criteria for frontotemporal dementia (FTD) [10, 11]. Cognitive impairment in people with ALS is characterised by executive dysfunction, impairments in language and social cognition [12–14], whilst apathy is the most pronounced behavioural change [15].

Individuals with chronic physical illness and comorbid depression or anxiety often experience a higher level of somatic symptom burden that can impair functioning [16]. Symptoms of depression, anxiety and apathy reduce quality of life (QoL) for people with ALS [17, 18]. Lower psychological well-being generally has been identified as predictive of disability and shorter survival in people with ALS [4]. Presence of concomitant neuropsychiatric conditions and cognitive impairment predicts greater carer distress [19–21].

Despite this, neuropsychiatric symptoms are often under-recognised in clinical care. Assessments are often performed using tools which have not been adapted for people with physical disability or communication difficulties. Assessments may also not be specifically addressing the cognitive domains impaired in people with ALS, or providing disease-specific thresholds for impairment. Scores may be significantly impacted by the overlap of somatic features of neuropsychiatric conditions and those attributable to the progression of ALS. In a group that is predominantly affected by motor impairment, speech and respiratory difficulties, lengthy administration time can also reduce the suitability of an tool.

### Assessment in clinical trials

The only globally licensed disease modifying therapy, riluzole [22], was introduced for use in people with ALS in the 1990s, following modest, but statistically significant prolongation of survival by two to three months [23–25]. Edaravone (Radicava) and masitinib have both emerged as promising treatments following positive trials in highly stratified cohorts [26], but neither have been licensed globally, owing to concerns around trial design and conduct. All of these current disease modifying therapies are intended to target the motor symptoms of ALS. To this end, research is underway to improve our understanding of the molecular mechanisms underlying ALS. This may in turn reveal potential important therapeutic targets, including those that modulate cognition [27, 28]. Cognitive assessments have been included in some studies investigating the effects of rilzuole as a disease modifying therapy in various other neurodegenerative conditions [29]. To our knowledge, the impact of riluzole on cognition in people with ALS has not been investigated in initial licensing trials.

There is an urgent need for more effective treatment options in ALS. Whilst survival and functioning remain the gold standard outcome measures used in clinical trials, incorporation of measures of neuropsychiatric and cognitive function enable more holistic assessment of the impact of drugs. Awareness of how a candidate drug may affect extra-motor features is clinically revelant in disease management and quality of life.

Including individuals with neuropsychiatric and cognitive impairments, and using assessments to evaluate changes in these areas as outcome measures will enable the trial teams to evaluate the impact of the candidate drugs on people with extra-motor features of ALS.

Candidate drugs may have a selective effect on neuropsychiatric or cognitive symptoms; therefore, measurement of these areas is pertinent to investigate the impact of an investigative medicinal product on both motor and extra-motor symptoms in ALS. The potential impact of candidate drugs is of therapeutic relevance, even if not the primary intended effect, considering the possible affect on quality of life and disease mangement of ALS.

Indeed, the 2019 revision of Airlie House consensus guidelines for design and implementation of ALS clinical trials [30] recommends that “Investigators may include assessments of cognitive or behavioral function as primary or secondary outcome measures”. Despite evolving guidance, and significant progess in establishing cognitive assessments within clinical care [31], we hypothesised that neuropsychiatric and cognitive symptoms have been under-evaluated in clinical trials and that when assessed using standardised tools, these tools are not specifically designed for people with ALS, which may affect their suitability for use in this population.

We aimed to systematically review 25 years of clinical trials in ALS, since the licensing of riluzole, to identify if neuropsychiatric symptoms and cognitive impairment were evaluated. If they were evaluated, we assessed if this was as an exclusion criteria or outcome measure and describe the tools used.

## Methods

We completed a systematic, unbiased, search of trial registries including clinicaltrials.gov, World Health Organisation’s (WHO) International Clinical Trials Registry Platform (ICTRP), European Union Clinical Trials Register (EduraCT) and PubMed on 18/11/2019 for Clinical Trials of an Investigational Medicinal Product (CTIMPs).

Using the search terms “amyotrophic lateral sclerosis” or “motor neuron* disease” we searched clinicaltrials.gov for interventional trials of investigative medicinal products. We searched European Union Clinical Trials Register (EduraCT) and WHO International Clinical Trials Registry Platform (ICTRP) for trials of “amyotrophic lateral sclerosis” with the filters “Phase II”, “Phase III” and “Phase IV” applied. Using the advanced search feature we filtered PubMed with [“amyotrophic lateral sclerosis”(MeSH Terms) OR “motor neuron* disease”(MeSH Terms)]. We then applied the ‘Clinical Trial’ filter for Article Type, Human trials only and Publication Date within the criteria defined above.

Phase II, III or IV trials assessing potential disease modifying therapies in subjects with amyotrophic lateral sclerosis that were registered, completed or published between 01/01/1994 to 31/10/2019 were included. No language restrictions were applied. Extension trials, post-hoc analysis papers and trials focused on symptom management were excluded.

### Data extraction

The following details of selected trials were extracted: “Investigative Medicinal Product (IMP) Assessed”, “Number of Participants”, “Date of Commencement”, “Primary Outcome Measure(s)” and “Secondary Outcome Measure(s)”.

Assessment of neuropsychiatric conditions or cognitive symptoms within each trial was categorised as follows: “Exclusion Criteria”, “Primary Outcome Measure”, “Secondary Outcome Measure”, “No Data Available for Trial” or “Not Assessed”. We also noted the assessment tool included, if documented, and a brief summary of the trial’s stipulations regarding neuropsychiatric conditions or cognitive symptoms. We also evaluated the use of Quality of Life measures within the trials. We investigated the number of clinical trials that recruited people with fronto-temporal dementia (FTD).

We documented the tools used and described the characteristics of tools used to evaluate neuropsychiatric or cognitive symptoms in trials identified in this systematic review. The areas assessed, features of administration and availability of disease-specific scores were recorded.

## Results

### Overview

**Fig. 1:**
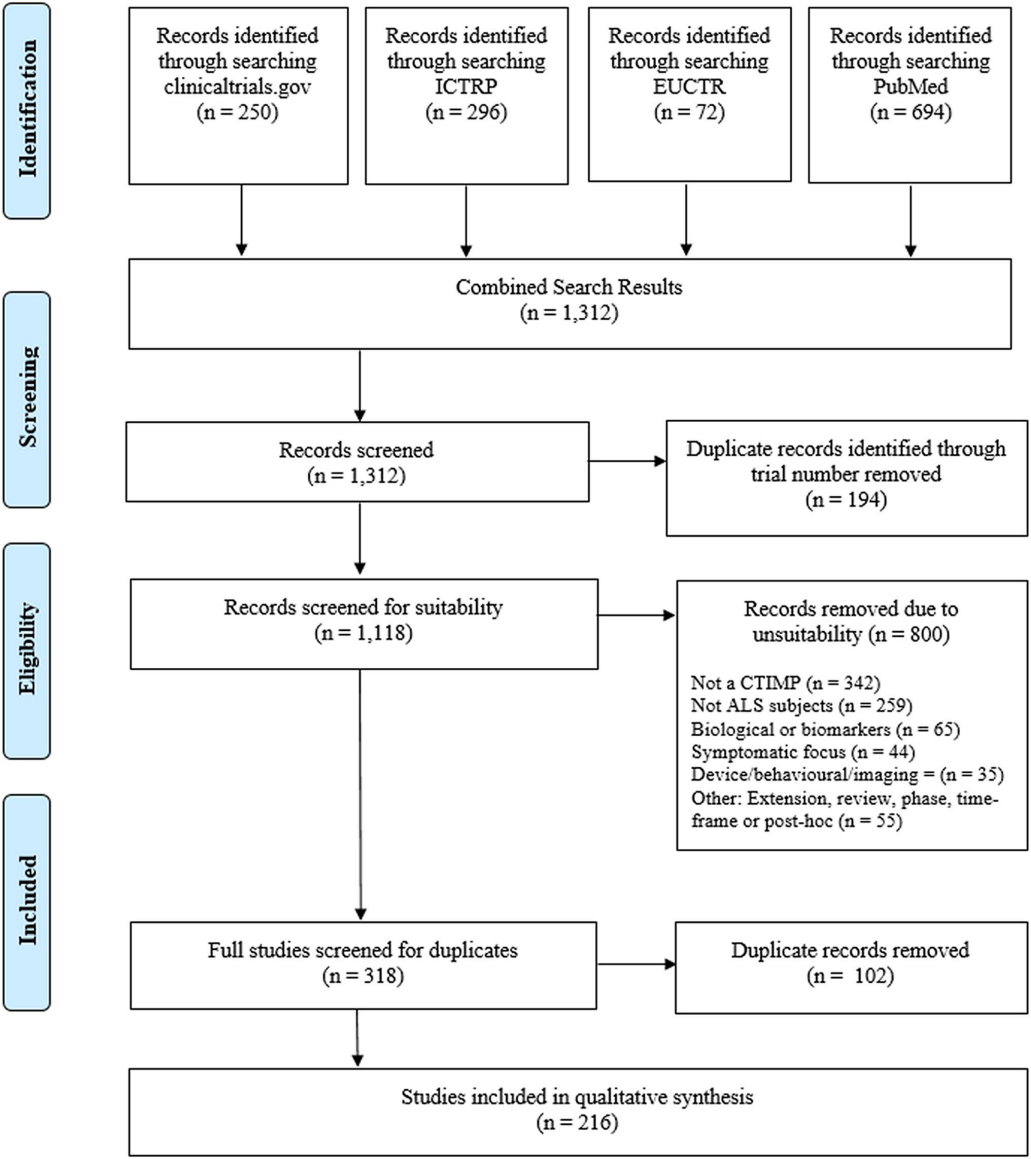
PRISMA Diagram for Record Selection. From Moher D, et al [71]. For more information, visit https://www.prisma-statement.org

A total of 1,312 records were identified (see PRISMA diagram in Fig. 1 for details). 296 duplicates were removed and a further 800 results were removed due to unsuitability (defined by inclusion criteria with full overview in Fig. 1); non-CTIMP and non-ALS subjects were the primary reasons for excluding search results. This resulted in 216 clinical trials of investigative medicinal products. 216 trials, proposed to include a total of 26,326 participants with ALS, were included in the final review.

Only one trial, the Therapy in Amyotrophic Lateral Sclerosis (TAME) trial evaluating memantine (Barohn et al., currently recruiting, Trial ID: NCT02118727), assessed both neuropsychiatric symptoms and cognitive impairment as Secondary Outcome Measures. A full list of the 13 trials assessing neuropsychiatric symptoms and cognitive impairment as primary or secondary outcome measures, the current status and results’ availability is shown in Table 1.

**Table 1:** Trials including neuropsychiatric and cognitive outcome measures

Unique trial identifier	Data source	Start date	Trial IMP	Neuropsychiatric assessment tool	Cognitive impairment assessment tool	Use of assessment as outcome measure	Status	Results summary
NCT00072709	Clinical Trials.gov	Sept 2003	Omigapil		Addenbrooke’s cognitive examination (ACE)	Secondary	Complete	No efficacy for IMP or change in neurocognitive measure
NCT00353665	Clinical Trials.gov	July 2005	Memantine & Riluzole	Hamilton depression (HAM-D)		Secondary	Complete	No efficacy for IMP and no results on HAM-D available
NCT00409721	Clinical Trials.gov	March 2007	Memantine		Addenbrooke’s cognitive examination (ACE)	Primary	Complete	No results available
2008–006,891-31	EudraCT	June 2009	Lithium carbonate	Hospital anxiety and depression scale (HADS)		Secondary	Complete	No efficacy for IMP or difference in HADS scores across groups
NCT02118727	Clinical Trials.gov	November 2011	Memantine	NeuroPsychiatric Inventory (NPI)	ALS Cognitive behavioral screen (ALS-CBS)	Secondary	Recruiting	
NCT01935518	Clinical Trials.gov	Sept 2013	Fasudil		Frontal behavior inventory (FBI) & verbal fluency	Secondary	Unknown	
NCT02868580	Clinical Trials.gov	October 2016	Triumeq	Columbia suicide severity rating scale (C-SSRS)		Secondary	Complete	IMP safe and potential efficiacy. Suicidal ideation in 2 participants, unrelated to IMP
NCT03508453	Clinical Trials.gov	August 2018	IC14		Edinburgh cognitive and behavioural ALS screen (ECAS)	Secondary	Withdrawn	
NCT03652805	Clinical Trials.gov	August 2018	IPL344	Hospital anxiety and depression scale (HADS) and ALS-depression-inventory (ADI-12)		Secondary	Recruiting	
NCT03293069	Clinical Trials.gov	January 2019	Deferiprone		Edinburgh cognitive and behavioural ALS screen (ECAS) and Montreal cognitive assessment (MoCA)	Secondary	Recruiting	
NCT03690791	Clinical Trials.gov	January 2019	CBD Oil		Edinburgh cognitive and behavioural ALS screen (ECAS)	Secondary	Recruiting	
KCT0001984	ICTRP	March 2019	Mecasin	Hamilton depression (HAM-D)		Secondary	Complete	No results available
NCT04082832	Clinical Trials.gov	Sept 2019	Cu(2ii)ATSM		Edinburgh cognitive and behavioural ALS screen (ECAS)	Primary	Recruiting	

Four trials had results available. In the trial of memantine (NCT00353665) the use of Hamilton depression scale as a planned secondary outcome measure was noted in the trial record on clinicaltrials.gov; however, there was no discussion of use or results of this outcome in the final report of the trial findings [32]. In a trial evaluating lithium carbonate (EudraCT 2008–006,891-31), depression worsened and anxiety increased over time in participants, but there was no significant difference between study groups [33].

A trial of omigapil (NCT00072709) reported no change in neurocognitive evaluations, utilising the Addenbrooke’s Cognitive Examination (ACE) [34]. The triumeq trial (NCT02868580) assessed suicidal ideation as a secondary outcome measure using the Columbia Suicide Severity Rating Scale (C-SSRS). Only two participants (5% of total trial participants) reported suicidal ideation and this was deemed unrelated to triumeq [35].

### Neuropsychiatric symptoms

35% (*n* = 76/216) of the total 216 trials included in this review assessed neuropsychiatric symptoms. Of these 76 trials, 92% (*n* = 70/76) assessments were used as an Exclusion Criteria, 3% (*n* = 2/76) as a Secondary Outcome Measure and 5% (*n* = 4/76) defined neuropsychiatric symptoms as both a Secondary Outcome Measure and an Exclusion Criterion.

29% (*n* = 22/76) of the 76 trials that reported assessing neuropsychiatric symptoms utilized investigator clinical judgement, 32% (*n* = 24/76) clinical history or medical records and 11% (*n* = 8/76) specified a standardized assessment tool.

### Cognitive impairment

21% (*n* = 46/216) of the 216 trials included assessed cognitive functioning. Of these 46 trials assessing cognitive functioning, 83% (*n* = 38/46) assessments were used as an Exclusion Criteria, 4% (*n* = 2/46) as a Primary Outcome Measure and 13% (*n* = 6/46) as a Secondary Outcome Measure.

Of these 46 trials which reported assessing cognitive impairment and provided data on the method of assessment, 23% (*n* = 11) utilized investigator clinical judgement, 9% (*n* = 4) clinical history or medical records and 26% (*n* = 12) specified a standardized assessment tool.

The Edinburgh Cognitive and Behavioural ALS Screen (ECAS) was the only assessment tool used as both a Primary and Secondary Outcome Measure to assess cognitive impairment in people with ALS.

A comorbid diagnosis of dementia was an Exclusion Criteria in 32% (*n* = 69) of the 216 trials reviewed. Subjects with dementia were explicitly referenced as able to participate in just 1% of trials (*n* = 3). 67% (*n* = 144) did not provide data on whether they accepted participants with a dementia diagnosis.

Quality of life measures were utilized as Primary and Secondary Outcome Measures in 1% (*n* = 3) and 27% (*n* = 59), respectively, of 216 trials reviewed. Table 2 summarises quality of life measures used and considers their content as some measures include limited assessments of mood and self-reported psychological health and functioning.

**Table 2:** Quality of life assessment tools and areas assessed

Assessment name	Total number of items	Number of trials utilised	Domains addressed	Mood items
Amyotrophic lateral sclerosis assessment questionnaire-40 Item (ALSAQ-40)	40	14	Hopelessness, depression and emotional reactivity	Ten items with 5-point Likert scale for frequency of mood symptoms
Amyotrophic lateral sclerosis assessment questionnaire-5 Item (ALSAQ-5)	5	6	Hopelessness	One item with 5-point Likert scale on frequency of hopelessness
ALS specific quality of life (ALSQOL-R)	50	6	Depression and anxiety	Ten-point Likert rating scales for level of agreement or frequency of experiences
EuroQol 5 domain assessment (EQ-5D-5L)	5	4	Health perception and functional impact	Five-point Likert rating scale for severity of anxiety/depression combined
Edmonton symptom assessment system (ESAS)	10	1	Pain, depression, anxious and general wellbeing	10-point Likert rating scale for depression and anxiety
McGill quality of life-revised	14	7	Depression, anxiety, mood and hopelessness	10-point Likert scale for severity of mood symptoms in previous 48 h
Schedule for the evaluation of individual quality of life questionnaire (SEIQoL)	3	1	Self-reported areas of concern and effect on function	Identify five most important areas in their life and rate importance
Short form patient questionnaire – 12 item (SF-12)	12	3	Emotional problems	Two items identifying the extent to which emotional problems affected activities
Short form patient questionnaire – 36 item (SF-36)	36	6	Low mood, lack of energy, anxiety	Four items identifying the extent to which emotional problems affected activities
Sickness impact profile – ALS Items (SIP/ALS-19)	19	1	ALS-adapted SIP Focus on activities of daily living, self-care and activities	No items specifically focused on mood, 1 item on social interaction
Visual analogue scale (VAS—3 health status questions)	3	1	General, physical and mental health self-rating	0–100 scale for overall mental health
Visual analogue scale and patient’s global impression of change	2	1	Pain and self-perception of health	7-point and 10-point Likert rating scales for change in emotion and overall quality of life

### Characteristics of assessment tools identified

The only tool specifically designed and validated for neuropsychiatric assessment in people with ALS, the ALS Depression Inventory (ADI-12) [36], was used in one trial. This tool is validated to screen for depressive symptoms in people with ALS, with a sensitivity of 100% and specificity of 82%, meaning all people with a major depressive disorder were identified [36]. However, this tool focuses only on the evaluation of depression and does not account for the range of neuropsychiatric symptoms which may affect people with ALS.

The other tools used in the trials included in this review are Beck’s Depression Inventory (BDI) [37], Hamilton Depression Inventory (HAM-D) [38], Columbia Suicide Severity Score (C-SSRS) [39], Hosptial Anxiety and Depression Scale (HADS) [40] and the Neuropsychiatric Inventory Questionnaire (NPI-Q) [41]. The BDI and HAM-D are extensively validated [42, 43], well-established measures of depressive symptoms. However, their reliance on somatic features of depression mean scores may be confounded by physical decline and no ALS-specific impairment thresholds are available. The HADS is designed to reduce the focus on somatic symptoms and ALS-specific impairment thresholds have been proposed [44].

The C-SSRS is considered a useful tool to structure discussions of suicidal ideation and intent [45]. However, the C-SSRS can be lengthy to administer, insufficient to cover the full spectrum of suicidal ideation [46] and responses to questions of end-of-life planning may be influenced by the presence of a terminal diagnosis. The NPI-Q is a validated informant-based questionnaire to evaluate the presence and severity of 12 neuropsychiatric domains [47].The ability of the tool to discriminate effectively between different disorders has been questioned [48].

Of the eight trials that did assess cognitive and behaviour change as an outcome measure, six used tools where scoring was unaffected by physical disability or communication impairment and were specifically designed, and validated, to identify domains impaired in people with ALS. One trial used the ALS Cognitive Behavioral Screen (ALS-CBS) [49] and five trials used the Edinburgh Cognitive and Behavioural ALS Screen (ECAS) [50].

The ALS-CBS is a brief assessment of executive functions while the ECAS assesses a wider profile of cognitive and behavioural impairment in ALS including executive and language functions, fluency and social cognition. Both are designed to be completed by either written or spoken responses to be suitable for ALS patients with differing functional ability. Additionally, ECAS scoring is also corrected for differences in motor speed during both speech and writing tasks, to accommodate for a range in disability severity.

Additionally, the Montreal Cognitive Assessment Scale (MoCA) [51], Addenbrooke’s Cognitive Examination (ACE-III) [52] and Mini Mental State Examination (MMSE) were used to evaluate cognition in the trials reviewed. Although widely validated for use in neurological conditions, particularly dementia, these tools were not specifically designed to assess the cognitive profile of people with ALS. As a result, no ALS-adapted thresholds for impairment were available and scoring on the tools was potentially impacted by motor decline experienced in ALS.

Verbal fluency is a commonly found deficit in ALS and is included as a sub-test on other cognitive assessments identified in this review and as a standalone test of cognition in one trial. However, it was not clear from the trial record if motor speed was controlled for in the assessment which can affect interpretation of the scores [12].

The Frontal Behavior Inventory (FBI) [53] and the behavioural interview of the ECAS and ALS-CBS were utilised to screen for behavioural symptoms which may be indicative of fronto-temporal dementia.

An additional assessment tool utilised by one trial, to exclude individuals with an intellectual disability, is the Weschler Adult Intelligence Scale (WAIS) [54]. As the WAIS was intend to screen for lower intelligence quotients rather than specific cognitive impairments we have not included this tool in Table 3 which focuses specifically on tools used to assess neuropsychiatric symptoms and cognitive impairment.

**Table 3:** Characteristics of tools used to evaluate cognition and neuropsychiatric symptoms

	Area assessed in trials	Domains	Designed for, or adapted for ALS with abnormality cut-offs	Administration time (min)	Total score	References
Beck’s depression inventory	Depression	Depression	−	5–10	63	[37]
Columbia suicide severity score	Suicidality	Suicidality	−	5–30	Binary outcome of 10 categories	[39]
Hamilton depression scale	Depression	Depression	−	20–30	52	[38]
Hospital anxiety and depression scale	Anxiety and depression	Anxiety and depression	+	5–10	21 per disorder	[40, 44]
Neuropsychiatric inventory questionnaire	Depression	Neuropsychiatric conditions	−	5	100 for 10-item & 144 for 12-item	[41, 47]
Amyotrophic lateral sclerosis depression inventory	Depression	Depression	+	5	48	[36, 66]
Amyotrophic lateral sclerosis cognitive behavioural screen	Cognition and behaviour	Executive functioning and behaviour	+	5–10	20 for cognitive section	[49, 61]
Addenbrooke’s cognitive examination	Cognition	Attention, memory, verbal fluency, language and visuospatial	−	15 +	100	[67]
Montreal cognitive assessment	Cognition	General cognition	−	10–15	30	[51, 68]
Edinburgh cognitive and behavioural assessment screen	Cognition and behaviour	Executive functioning, language, fluency, memory, visuospatial, social cognition, verbal fluency index and behaviour	+	15–25	136 for cognitive section	[50]
Frontal behavioural inventory	Behaviour	Behaviours characteristic of FTD	−	15–30	Severity rating 0–3 on 24 items	[53, 69]
Verbal fluency	Cognition	Language	+	< 5	N/A	[12, 53]
Mini mental state examination	Cognition	General cognition	−	< 5	30	[70]

+ Test designed or adapted for ALS and ALS-specific impairment thresholds are available

−Test not designed/adapted for ALS and no ALS-specific impairment thresholds currently available

*NB Time to administer is indicated as a guide only. This will vary depending upon the respondent’s functional ability, presence of a communication impairment and use of assistive devices and the researcher’s experience in administering the instrument*

## Discussion

This review of 216 clinical trials from the last 25 years of ALS research highlights that the assessment of neuropsychiatric and cognitive symptoms has been frequently neglected. Only one trial assessed the impact of the candidate drug on both neuropsychiatric symptoms and cognitive impairment. This is despite overwhelming evidence that these extra-motor features are prevalent across, and impactful upon, people with ALS [5, 9, 20].

Whilst the impact on physical functioning and survival justifiably remain the primary objectives for clinical trials of ALS, the importance of additional assessments for neuropsychiatric and cognitive functioning needs to be addressed. This will enable future trial design to align with the 2019 revision of Airlie House consensus guidelines, recommendations for the design and implementation of clinical trials in ALS [30].

As the only globally licensed treatment for ALS, riluzole use is sometimes an exclusion criteria, or a minimisation criteria at point of randomisation, in many ALS trials. The effect of riluzole on cognition in people with ALS is not yet established, with study results reporting variable impact [56, 57]. Frequent inclusion in trials in other neurological conditions, and conflicting findings, provides further justification for the inclusion of cognitive measures to evaluate how established medications such as riluzole, and exploratory study drugs, impact upon people with ALS.

### Assessment of neuropsychiatric symptoms in clinical trials

Despite research indicating the presence of neuropsychiatric symptoms and comorbidities as pervasive [3, 55] and pernicious [56] in people with ALS, the impact of candidate drugs on neuropsychiatric functioning is underevaluated. Of the 216 trials included in this review, only eight reported using a formal neuropsychiatric assessment tool. Accuracy of evaluation is additionally hindered by the limited availability of tools specifically designed and validated for neuropsychiatric evaluation of the ALS population. To evaluate the impact of candidate drugs on neuropsychiatric domains potentially affected in ALS, without significant confounding of physical disability, a greater understanding of the validity of these measures in ALS trials is needed.

### Assessment of cognition in clinical trials

The past 15 years of research has highlighted the ubiquity of cognitive and behavioural impairment in people with ALS, [50, 57]. Whilst the assessment of these changes has become widely accepted in clinical care for people with ALS [58], clinical trials are lagging behind, with only 21% of the 216 trials reviewed in this study assessing cognition.

The inclusion of cognitive assessment in clinical trial design as an additional outcome measure is progressing slightly. In the previous decade of research, 2010–2020 inclusive, 7% (*n* = 6) of 86 trials registered, commenced or published within this time frame assessed cognition as an outcome measure. In comparison, of 101 trials from the decade prior, 2000–2010, 2% (*n* = 2) assessed cognition as an outcome measure.

Cognitive assessment in trial design is also increasing in accuracy, in part due to the availability of ALS-specific tools validated specifically to assess the domains impaired in this cohort [49, 59], particularly the ALS Cognitive Behavioral Screen (ALS-CBS) and Edinburgh Cognitive and Behavioural ALS Screen (ECAS) in the trials reviewed here. Cognitive assessments used previously may not be suitable for individuals with physical disabilities, due to an over-reliance on motor tasks, and limited focus on cognitive domains impaired in people with ALS.

Our review focused on the inclusion of cognitive functioning assessment in trial design; however, assessing evolution in in cognitive functioning during disease trajectory may also be of interest in future trial design. Longitudinal cognitive assessments were evaluated as an outcome measure in the ALS Multicenter Cohort Study of Oxidative Stress (ALS COSMOS [60]). Baseline data in this study of 247 participants evaluated with the ALS-CBS indicated that cognitive and behavioural impairments were common, 6.5% scoring below cut-offs for frontotemporal dementia [49], 54.2% scoring consistent with mild cognitive impairment and behavioural subscores outwith the normal range in 30.6% of responders [61]. Analysis of longitudinal data did not detect cognitive decline over a 12-month period but did detect an increase in behavioural changes, notably disinhibition, while initial behavioural impairment predicted attrition [62]. Other studies have demonstrated increasing prevalence of cognitive and behavioural impairment in later disease stages [63, 64].

Non-interventional studies which have assessed change in cognition over time have shown mixed results [64, 65]. Attrition of individuals with cognitive and/or behavioural impairments from longituinal repeated assessments may also bias the sample towards individuals with slower disease progression and more stable cognitive functions.

Only one trial in this review, the Omigapil trial (NCT00072709), reported data on cognitive otucomes. No change in neurocognitive secondary outcome measure was reported, with the Addenbrooke’s Cognitve Examination (ACE) [34]. Understanding of cognitive change across the disease course is of prime importance for the interpretation of cognitive data in clinical trials.

### Impact on generalisability of trial findings

A diagnosis, historical or current, of symptoms of a neuropsychiatric disorder was still explicitly identified as an exclusion criterion for the majority of trials included in this study.

Cognitive impairment was also often included as an exclusion criterion, with thresholds for impairment often subjective or not addressed. This could be due to researchers’ concerns over capacity to consent, potential issues with trial adherence, compliance with assessments and following medication regimens.

Ultimately, results from studies with strict exclusion criteria, which reduce the sample representativeness of a heterogenous patient group, may not be generalisable to the whole ALS population. Furthermore, excluding individuals with neuropsychiatric conditions and cognitive impairment, unless when absolutely necessary, and neglecting to include these aspects as outcome measures, misses a vital opportunity to explore how candidate drugs may affect people with ALS across this diverse condition.

## Conclusion

This study clearly demonstrates that the impact of candidate drugs on neuropsychiatric symptoms and cognition has been under-evaluated in clinical trials and that when these symptoms have been evaluated, the tools used may not be suitable for people with ALS.

Accurate identification of neuropsychiatric comorbidity and cognitive impairment is an essential requirement to improve our understanding of how candidate drugs impact the extra-motor features of ALS.

We recommend the evaluation of neuropsychiatric and cognitive symptoms as additional outcome measures in clinical trials of investigative medicinal products. Additionally, when evaluating these areas we recommend using tools which are designed to assess domains affected in ALS, with disease-specific impairment thresholds and those adapted to account for motor decline and communication difficulties.

## Electronic supplementary material

Below is the link to the electronic supplementary material.

Supplementary file 1 (DOCX 122 kb)

## Data Availability

Not applicable.
