# Supplementary material for: A systematic review of neuropsychiatric and cognitive assessments used in clinical trials for amyotrophic lateral sclerosis
Source: J Neurol. 2020 Sep 10;268(12):4510–21. doi: 10.1007/s00415-020-10203-z (PMC8563523; doi:10.1007/s00415-020-10203-z)
Supplement: Supplementary file 1 — (DOCX 122 kb) [file 415_2020_10203_MOESM1_ESM.docx]

| Supplementary Material Document 1 | | | | | | | | | | | | | | | | | | | | | | | | | | | | | | | | | | | | | |
| --- | --- | --- | --- | --- | --- | --- | --- | --- | --- | --- | --- | --- | --- | --- | --- | --- | --- | --- | --- | --- | --- | --- | --- | --- | --- | --- | --- | --- | --- | --- | --- | --- | --- | --- | --- | --- | --- |
| **ICTRIP Trial ID** | **NCT Number (Clinical Trials.Gov)** | | **EudraCT Trial ID** | | **PubMed ID** | | **Trial Number** | | **Title** | | **Phase** | | **Subject Number** | | **Start Date** | | **QoL Assessed?** | | **QoL Tool Used** | | **QoL Inclusion** | | **Neuropsychiatric Assessed?** | | **Neuropsychiatric  Tool Used** | | **Neuropsychiatric Inclusion?** | | **Accepting PwDementia** | | **Informed Consent Requirement** | | **Cognition Assessed?** | | **Cognition Assessment Tool** | | **Cognition Inclusion?** |
|  | NCT00072709 | |  | |  | | 122 | | Study Evaluating TCH346 and Placebo Administered Once Daily in Patients With Amyotrophic Lateral Sclerosis (ALS) | | Phase 2 | | 551 | | 01/09/2003 | | ND | | N/A | | N/A | | ND | | N/A | | N/A | | No | | Yes | | Yes | | ACE | | SOM |
|  | NCT00409721 | |  | |  | | 56 | | The Effect of Memantine on Functional Outcomes and Motor Neuron Degeneration in Amyotrophic Lateral Sclerosis (ALS) | | Phase 2 | | 42 | | 01/03/2007 | | ND | | N/A | | N/A | | Yes | | ND | | EC | | No | | ND | | Yes | | ACE | | POM |
|  | NCT02118727 | |  | |  | | 83 | | Therapy in Amyotrophic Lateral Sclerosis (TAME) | | Phase 2 | | 90 | | 07/11/2018 | | ND | | N/A | | N/A | | Yes | | Neuropsychiatric Inventory Questionnaire | | SOM | | ND | | Yes | | Yes | | ALS-CBS | | SOM |
|  | NCT03690791 | |  | |  | | 38 | | Efficacy of Cannabinoids in Amyotrophic Lateral Sclerosis or Motor Neurone Disease | | Phase 3 | | 30 | | 09/01/2019 | | Yes | | ALSQOL-R | | SOM | | Yes | | History | | EC | | ND | | Yes | | Yes | | ECAS | | SOM |
|  | NCT03508453 | |  | |  | | 63 | | IC14 for Treatment of Amyotrophic Lateral Sclerosis | | Phase 2 | | 50 | | 15/08/2018 | | Yes | | ALSSQOL-R | | SOM | | Yes | | History | | EC | | ND | | Yes | | Yes | | ECAS | | SOM |
|  | NCT04082832 | |  | |  | | 79 | | CuATSM Compared With Placebo for Treatment of ALS/MND | | Phase 2\|Phase 3 | | 80 | | 30/09/2019 | | ND | | N/A | | N/A | | ND | | N/A | | N/A | | ND | | Yes | | Yes | | ECAS | | POM |
|  |  | | 2014-005367-32 | | PMID:28801400 \| PMCID:PMC5724081 | | 168 | | Protein misfolding, amyotrophic lateral sclerosis and guanabenz: protocol for a phase II RCT with futility design (ProMISe trial). | | Phase 3 | | 208 | | ND | | ND | | N/A | | N/A | | ND | | N/A | | N/A | | ND | | Yes | | Yes | | ECAS | | EC |
|  | NCT01935518 | |  | |  | | 5 | | A Clinical Trial of Safety and Efficacy of Fasudil in Subjects With Amyotrophic Lateral Sclerosis (ALS) | | Phase 2 | | 10 | | 01/09/2013 | | Yes | | SF-36 | | SOM | | ND | | N/A | | N/A | | ND | | Yes | | Yes | | Frontal Behavioural Inventory & Verbal Fluency | | SOM |
|  | NCT01786174 | |  | | PMID:28662296 \| PMCID:PMC5724488 | | 39 | | Gilenya in Amyotrophic Lateral Sclerosis (ALS) | | Phase 2 | | 30 | | 01/08/2013 | | ND | | N/A | | N/A | | Yes | | History | | EC | | No | | ND | | Yes | | History | | EC |
|  | NCT02450552 | |  | |  | | 72 | | Clinical Trial of Ezogabine (Retigabine) in ALS Subjects | | Phase 2 | | 65 | | 01/06/2015 | | ND | | N/A | | N/A | | Yes | | History | | EC | | No | | ND | | Yes | | History | | EC |
|  | NCT02487407 | |  | |  | | 73 | | Effects of ODM-109 on Respiratory Function in Patients With Amyotrophic Lateral Sclerosis | | Phase 2 | | 66 | | 01/07/2015 | | Yes | | ND | | SOM | | Yes | | History | | EC | | No | | ND | | Yes | | History | | EC |
|  | ND | | 2007-002379-16 | |  | | 131 | | MULTICENTER TRIAL (SINGLE BLIND) VS ON THE EFFICACY OF LITHIUM SALTS +RILUZOLE VS PLACEBO+RILUZOLE IN PATIENTS AFFECTED BY AMYOTROPHIC LATERAL SCLEROSIS | | Phase 4 | | 100 | | 29/05/2007 | | ND | | N/A | | N/A | | Yes | | History | | EC | | ND | | Yes | | Yes | | History | | EC |
|  | NCT02714036 | |  | |  | | 44 | | A Biomarker Study to Evaluate MN-166 (Ibudilast) in Subjects With Amyotrophic Literal Sclerosis (ALS) | | Phase 1\|Phase 2 | | 35 | | 01/03/2016 | | ND | | N/A | | N/A | | Yes | | C-SSRS & Investigator Judgement | | EC | | No | | Yes | | Yes | | Investigator Judgement | | EC |
|  | NCT01906658 | |  | |  | | 57 | | A Study to Explore the Safety and Tolerability of Acthar in Patients With Amyotrophic Lateral Sclerosis | | Phase 2 | | 43 | | 01/07/2013 | | ND | | N/A | | N/A | | Yes | | History | | EC | | ND | | Yes | | Yes | | Investigator Judgement | | EC |
|  | NCT00748501 | |  | |  | | 60 | | Clinical Trial of SB-509 in Subjects With Amyotrophic Lateral Sclerosis (ALS) | | Phase 2 | | 45 | | 01/09/2008 | | ND | | N/A | | N/A | | Yes | | History | | EC | | No | | ND | | Yes | | Investigator Judgement | | EC |
|  | NCT03168711 | |  | |  | | 35 | | Safety of Urate Elevation in Amyotrophic Lateral Sclerosis (ALS) | | Phase 2 | | 30 | | 01/10/2017 | | ND | | N/A | | N/A | | Yes | | Investigator Judgement | | EC | | No | | ND | | Yes | | Investigator Judgement | | EC |
|  | NCT03427086 | |  | |  | | 36 | | Safety and Tolerability of High Dose Biotin in Patients With Amyotrophic Lateral Sclerosis | | Phase 2 | | 30 | | 29/01/2018 | | ND | | N/A | | N/A | | Yes | | Investigator Judgement | | EC | | No | | ND | | Yes | | Investigator Judgement | | EC |
|  |  | |  | | PMID:22323869 \| PMCID:PMC3271295 | | 172 | | Oral solubilized ursodeoxycholic acid therapy in amyotrophic lateral sclerosis: a randomized cross-over trial. | | ND | | 63 | | ND | | ND | | N/A | | N/A | | Yes | | Investigator Judgement | | EC | | No | | Yes | | Yes | | Investigator Judgement | | EC |
|  | NCT01806857 | |  | | PMID:28070747 \| PMCID:PMC5509619 | | 82 | | Clinical Trial Nuedexta in Subjects With ALS | | Phase 2 | | 90 | | 01/04/2013 | | ND | | N/A | | N/A | | Yes | | Investigator Judgement | | EC | | No | | ND | | Yes | | Investigator Judgement | | EC |
|  | NCT03272503 | |  | | PMID:29202456 | | 84 | | A Clinical Trial of Pimozide in Patients With Amyotrophic Lateral Sclerosis (ALS) | | Phase 2 | | 100 | | 27/10/2017 | | Yes | | ALSQOL-R | | SOM | | Yes | | Investigator Judgement | | EC | | No | | ND | | Yes | | Investigator Judgement | | EC |
|  | NCT03127514 | |  | |  | | 92 | | AMX0035 in Patients With Amyotrophic Lateral Sclerosis (ALS) | | Phase 2 | | 132 | | 22/06/2017 | | ND | | N/A | | N/A | | Yes | | Investigator Judgement | | EC | | No | | ND | | Yes | | Investigator Judgement | | EC |
|  | NCT02496767 | |  | |  | | 125 | | Ventilatory Investigation of Tirasemtiv and Assessment of Longitudinal Indices After Treatment for a Year | | Phase 3 | | 743 | | 03/09/2015 | | ND | | N/A | | N/A | | Yes | | Investigator Judgement | | EC | | ND | | Yes | | Yes | | Investigator Judgement | | EC |
| JPRN-UMIN000022050 | |  | |  | | 152 | | A Phase II, Randomised, Double-Blind, Placebo-Controlled Study Followed by a Continuing Open Label Study, to Verify the Efficacy and Safty of Intrathecal Administration of KP-100IT using NP022 in Subjects with Amyotrophic Lateral Sclerosis (ALS) | | ND | | 48 | | ND | | Yes | | ALSAQ40 | | SOM | | Yes | | Investigator Judgement | | EC | | No | | ND | | Yes | | Investigator Judgement | | EC | |
|  | NCT00353665 | | 2007-002117-39 | | PMID:20565333 | | 70 | | Memantine for Disability in Amyotrophic Lateral Sclerosis (MEDALS) | | Phase 2\|Phase 3 | | 63 | | 01/07/2005 | | Yes | | SF-36 | | SOM | | Yes | | Hamilton Depression | | Mixed | | No | | ND | | Yes | | MMSE | | EC |
|  | NCT00140218 | |  | |  | | 41 | | R(+) Pramipexole in Early Amyotrophic Lateral Sclerosis | | Phase 1\|Phase 2 | | 30 | | 01/08/2005 | | ND | | N/A | | N/A | | Yes | | ND | | EC | | No | | ND | | Yes | | MMSE | | EC |
|  | NCT03293069 | |  | |  | | 106 | | Conservative Iron Chelation as a Disease-modifying Strategy in Amyotrophic Lateral Sclerosis | | Phase 2\|Phase 3 | | 240 | | 30/01/2019 | | Yes | | ALSAQ-5 | | SOM | | Yes | | Investigator Judgement | | EC | | No | | ND | | Yes | | MoCA & ECAS | | SOM |
|  | NCT00035815 | |  | | PMID:19029516 | | 110 | | Insulin-like Growth Factor-1 in Amyotrophic Lateral Sclerosis (ALS) Trial | | Phase 3 | | 330 | | 01/06/2003 | | ND | | N/A | | N/A | | Yes | | Beck's Depression Inventory | | EC | | ND | | ND | | ND | | N/A | | N/A |
|  | NCT02868580 | |  | |  | | 58 | | Safety and Tolerability of Antiretroviral (Triumeq) in Patients With Amyotrophic Lateral Sclerosis (ALS). | | Phase 2 | | 43 | | 01/10/2016 | | ND | | N/A | | N/A | | Yes | | C-SSRS & History | | Mixed | | No | | Yes | | ND | | N/A | | N/A |
| ISRCTN83178718 | ND | | 2008-006891-31 | | PMID:23453347\|PMID:21936930 | | 137 | | A randomised placebo-controlled trial of Lithium carbonate in Amyotrophic Lateral Sclerosis (LiCALS) | | Phase 4 | | 220 | | 17/02/2009 | | Yes | | EQ-5D-5L | | SOM | | Yes | | HADS | | SOM | | ND | | ND | | ND | | N/A | | N/A |
|  | NCT03652805 | |  | |  | | 12 | | A Study of IPL344 in the Treatment of ALS Patients | | Phase 1\|Phase 2 | | 15 | | 01/08/2018 | | ND | | N/A | | N/A | | Yes | | HADS & ADI-12 | | Mixed | | ND | | Yes | | ND | | N/A | | N/A |
| KCT0001984 |  | |  | |  | | 153 | | a phase II double-blind randomized placebo-controlled trial of combined Mecasin and riluzole treatment in patients with amyotrophic lateral sclerosis | | ND | | 36 | | 01/03/2019 | | Yes | | Visual Analogue Scale PGIC | | SOM | | Yes | | Hamilton Depression | | Mixed | | No | | Yes | | ND | | N/A | | N/A |
|  | NCT04140136 | |  | |  | | 14 | | The Efficacy and Safety of Vitamin E Mixed Tocotrienols In Patients With Amyotrophic Lateral Sclerosis (ALS) | | Phase 2 | | 20 | | 17/06/2019 | | ND | | N/A | | N/A | | Yes | | History | | EC | | ND | | Yes | | ND | | N/A | | N/A |
|  | NCT00925847 | |  | |  | | 27 | | Effect of Lithium Carbonate in Patients With Amyotrophic Lateral Sclerosis | | Phase 2 | | 23 | | 01/06/2009 | | ND | | N/A | | N/A | | Yes | | History | | EC | | No | | Yes | | ND | | N/A | | N/A |
|  | NCT00415519 | |  | | PMID:28872919 | | 32 | | Efficacy and Safety Study of MCI-186 for Treatment of Amyotrophic Lateral Sclerosis (ALS) Who Met Severity Classification III | | Phase 3 | | 25 | | 01/12/2006 | | ND | | N/A | | N/A | | Yes | | History | | EC | | No | | ND | | ND | | N/A | | N/A |
|  | NCT02709330 | |  | |  | | 66 | | ALS Reversals - Lunasin Regimen | | Phase 2 | | 60 | | 01/04/2016 | | ND | | N/A | | N/A | | Yes | | History | | EC | | ND | | Yes | | ND | | N/A | | N/A |
|  | NCT02238626 | |  | |  | | 75 | | Ibudilast (MN-166) in Subjects With Amyotrophic Lateral Sclerosis (ALS) | | Phase 2 | | 71 | | 01/09/2014 | | Yes | | ALSAQ-5 | | SOM | | Yes | | History | | EC | | No | | Yes | | ND | | N/A | | N/A |
| ACTRN12608000338369 | |  | | PMID:26844270 | | 148 | | The effect of flecainide on the Amyotrophic Lateral Sclerosis Functional Rating Scale-revised in patients with amyotrophic lateral sclerosis | | Phase 2 | | 100 | | 01/07/2002 | | ND | | N/A | | N/A | | Yes | | History | | EC | | No | | ND | | ND | | N/A | | N/A | |
|  | NCT01492686 | |  | | PMID:28872919\|PMID:28872913\|PMID:28522181 | | 94 | | Phase 3 Study of MCI-186 for Treatment of Amyotrophic Lateral Sclerosis | | Phase 3 | | 137 | | 01/12/2011 | | Yes | | ALSAQ-40 | | SOM | | Yes | | History | | EC | | No | | ND | | ND | | N/A | | N/A |
|  | NCT03127267 | |  | |  | | 119 | | Efficacy and Safety of Masitinib Versus Placebo in the Treatment of ALS Patients | | Phase 3 | | 495 | | 01/11/2019 | | Yes | | ALSAQ-40 | | SOM | | Yes | | History | | EC | | No | | ND | | ND | | N/A | | N/A |
|  | NCT00868166 | |  | | PMID:24447620 | | 120 | | Safety and Efficacy of TRO19622 as add-on Therapy to Riluzole Versus Placebo in Treatment of Patients Suffering From ALS | | Phase 3 | | 512 | | 30/04/2009 | | Yes | | McGill | | SOM | | Yes | | History | | EC | | No | | Yes | | ND | | N/A | | N/A |
|  | NCT01709149 | |  | | PMID:26982815 | | 124 | | Study of Safety, Tolerability & Efficacy of CK-2017357 in Amyotrophic Lateral Sclerosis (ALS) | | Phase 2 | | 711 | | 01/10/2012 | | ND | | N/A | | N/A | | Yes | | History | | EC | | ND | | Yes | | ND | | N/A | | N/A |
|  | NCT01281189 | |  | | PMID:28178599\|PMID:27677562\|PMID:27385750\|PMID:25125035 | | 126 | | Phase 3 Study of Dexpramipexole in ALS | | Phase 3 | | 943 | | 01/03/2011 | | Yes | | ALSAQ-5 | | SOM | | Yes | | History | | EC | | ND | | ND | | ND | | N/A | | N/A |
| ChiCTR-IPR-15007365 | |  | |  | | 150 | | A Multi-center, Randomized, Double Blinding, Placebo-Controlled Clinical Trial of Dl-3-Butylphthalide in the Treatment of Amyotrophic Lateral Sclerosis | | ND | | 147 | | ND | | ND | | N/A | | N/A | | Yes | | History | | EC | | No | | Yes | | ND | | N/A | | N/A | |
| KCT0003792 |  | |  | |  | | 154 | | Double-eye, random assignment, and parallel 2b exploratory clinical trials to assess the effectiveness and safety of Mecasin and standard treatment (Riluzole) therapy in patients with ALS. | | ND | | 50 | | ND | | ND | | N/A | | N/A | | Yes | | History | | EC | | No | | Yes | | ND | | N/A | | N/A |
|  | ND | | 2015-001431-20 | |  | | 144 | | A randomized, double blind, double-dummy placebo controlled, 3-way cross-over study to determine the test-retest reliability of, and the effect of oral retigabine and riluzole | | Phase 2 | | 18 | | 17/08/2015 | | ND | | N/A | | N/A | | Yes | | Investigator Judgement | | EC | | No | | Yes | | ND | | N/A | | N/A |
|  | NCT00800501 | |  | |  | | 13 | | A Safety and Tolerability Study of Intracerebroventricular Administration of sNN0029 to Patients With Amyotrophic Lateral Sclerosis | | Phase 1\|Phase 2 | | 18 | | 01/12/2008 | | Yes | | EQ-5D-5L | | SOM | | Yes | | Investigator Judgement | | EC | | No | | Yes | | ND | | N/A | | N/A |
|  | NCT04066244 | |  | |  | | 23 | | Study of Safety and of the Mechanism of BLZ945 in ALS Patients | | Phase 2 | | 20 | | 23/10/2019 | | ND | | N/A | | N/A | | Yes | | Investigator Judgement | | EC | | No | | Yes | | ND | | N/A | | N/A |
|  | NCT01257581 | |  | |  | | 69 | | Safety and Efficacy Study of Creatine and Tamoxifen in Volunteers With Amyotrophic Lateral Sclerosis (ALS) | | Phase 2 | | 60 | | 01/03/2011 | | ND | | N/A | | N/A | | Yes | | Investigator Judgement | | EC | | ND | | Yes | | ND | | N/A | | N/A |
|  | ND | | 2008-006722-34 | |  | | 136 | | Multicentric randomized placebo controlled trial of Lithium carbonate combined with Riluzole in patients with ALS | | Phase 3 | | 226 | | 23/06/2009 | | Yes | | McGill | | SOM | | Yes | | Investigator Judgement | | EC | | No | | ND | | ND | | N/A | | N/A |
|  | NCT01879241 | |  | | PMID:29934198 | | 107 | | Study of Rasagiline in Patients With Amyotrophic Lateral Sclerosis | | Phase 2 | | 252 | | 01/06/2013 | | Yes | | SEIQoL | | SOM | | Yes | | Investigator Judgement | | EC | | No | | Yes | | Yes | | N/A | | N/A |
|  |  | |  | | PMID:11465936 | | 190 | | A double-blind, placebo-controlled randomized clinical trial of alpha-tocopherol (vitamin E) in the treatment of amyotrophic lateral sclerosis. ALS riluzole-tocopherol Study Group. | | ND | | 289 | | 01/06/1997 | | Yes | | SIP | | SOM | | Yes | | Investigator Judgement | | EC | | No | | Yes | | ND | | N/A | | N/A |
|  | NCT03505021 | |  | |  | | 117 | | Effects of Oral Levosimendan (ODM-109) on Respiratory Function in Patients With ALS | | Phase 3 | | 450 | | 21/06/2018 | | ND | | N/A | | N/A | | Yes | | Investigator Judgement | | EC | | ND | | Yes | | ND | | N/A | | N/A |
| ACTRN12618000534280 | |  | |  | | 149 | | Phase 2 Randomised Placebo Controlled Double Blind Study to Assess the Efficacy and Safety of Tecfidera in Patients with Amyotrophic Lateral Sclerosis (TEALS Study) | | Phase 2 | | 90 | | ND | | Yes | | ALSQOL-R | | SOM | | Yes | | Investigator Judgement | | EC | | No | | ND | | ND | | N/A | | N/A | |
|  | NCT03457753 | |  | |  | | 2 | | Riluzole Oral Soluble Film Safety and Tolerability in Amyotrophic Lateral Sclerosis | | Phase 2 | | 0 | | 01/03/2018 | | ND | | N/A | | N/A | | ND | | N/A | | N/A | | ND | | Yes | | ND | | N/A | | N/A |
|  |  | |  | | PMID:18335482 | | 164 | | Pilot study of granulocyte colony stimulating factor (G-CSF)-mobilized peripheral blood stem cells in amyotrophic lateral sclerosis (ALS). | | ND | | 8 | | ND | | ND | | N/A | | N/A | | ND | | N/A | | N/A | | ND | | ND | | ND | | N/A | | N/A |
|  |  | |  | | PMID:21254083\|PMID:19878077 | | 218 | | Pilot study of granulocyte colony stimulating factor (G‐CSF)‐mobilized peripheral blood stem cells in amyotrophic lateral sclerosis (ALS) | | ND | | 8.00 | | ND | | ND | | N/A | | N/A | | ND | | N/A | | N/A | | ND | | Yes | | ND | | N/A | | N/A |
|  | NCT03679975 | |  | |  | | 4 | | Riluzole Oral Soluble Film Swallowing Safety in Amyotrophic Lateral Sclerosis | | Phase 2 | | 9 | | 04/04/2018 | | ND | | N/A | | N/A | | ND | | N/A | | N/A | | ND | | Yes | | ND | | N/A | | N/A |
|  |  | |  | | PMID:8791245 | | 182 | | A double-blind placebo-controlled study of 3,4-diaminopyridine in amytrophic lateral sclerosis patients on a rehabilitation unit. | | ND | | 9 | | ND | | ND | | N/A | | N/A | | ND | | N/A | | N/A | | ND | | ND | | ND | | N/A | | N/A |
|  | NCT0081285 | |  | | PMID:22594565 | | 193 | | Single-dose pharmacokinetics and tolerability of oral delta-9- tetrahydrocannabinol in patients with amyotrophic lateral sclerosis. | | ND | | 9 | | 01/11/2007 | | ND | | N/A | | N/A | | ND | | N/A | | N/A | | ND | | Yes | | ND | | N/A | | N/A |
|  | NCT01259050 | |  | |  | | 6 | | Safety Study of High Doses of Zinc in ALS Patients | | Phase 1\|Phase 2 | | 10 | | 01/10/2010 | | ND | | N/A | | N/A | | ND | | N/A | | N/A | | ND | | Yes | | ND | | N/A | | N/A |
|  | NCT03506425 | |  | |  | | 7 | | A Pilot Trial of Triheptanoin for People With Amyotrophic Lateral Sclerosis (PALS) | | Phase 1\|Phase 2 | | 10 | | 21/06/2018 | | ND | | N/A | | N/A | | ND | | N/A | | N/A | | ND | | Yes | | ND | | N/A | | N/A |
|  | NCT00600873 | |  | |  | | 8 | | R(+)PPX High Dose Treatment of ALS | | Phase 1\|Phase 2 | | 10 | | 01/08/2007 | | ND | | N/A | | N/A | | ND | | N/A | | N/A | | ND | | ND | | ND | | N/A | | N/A |
| JPRN-UMIN000016352 | |  | |  | | 151 | | The Safety of Edaravone for patients with Amyotrophic Lateral Sclerosis: Single centered open label trial | | Phase 1/2 | | 10 | | ND | | ND | | N/A | | N/A | | ND | | N/A | | N/A | | ND | | ND | | ND | | N/A | | N/A | |
|  | NCT01854294 | |  | |  | | 9 | | GM604 Phase 2A Randomized Double-blind Placebo Controlled Pilot Trial in Amyotrophic Lateral Disease (ALS) | | Phase 2 | | 12 | | 01/08/2013 | | ND | | N/A | | N/A | | ND | | N/A | | N/A | | ND | | Yes | | ND | | N/A | | N/A |
|  |  | |  | | PMID:10227642 | | 192 | | The pharmacokinetics and pharmaco-dynamics of Procysteine in amyotrophic lateral sclerosis. | | ND | | 13 | | ND | | ND | | N/A | | N/A | | ND | | N/A | | N/A | | ND | | ND | | ND | | N/A | | N/A |
|  | NCT03613571 | | 2017-005065-47 | |  | | 10 | | A Study to Evaluate the Safety, Tolerability and Efficacy of ILB in Patients With Amyotrophic Lateral Sclerosis | | Phase 2 | | 13 | | 15/08/2018 | | Yes | | VAS - 3 health status questions | | SOM | | ND | | N/A | | N/A | | ND | | Yes | | ND | | N/A | | N/A |
|  |  | |  | | PMID:19935406 | | 166 | | Safety, tolerability, and cerebrospinal fluid penetration of ursodeoxycholic Acid in patients with amyotrophic lateral sclerosis. | | ND | | 18 | | ND | | ND | | N/A | | N/A | | ND | | N/A | | N/A | | ND | | ND | | ND | | N/A | | N/A |
|  | NCT01277315 | |  | | PMID:26444282 \| PMCID:PMC4596620 | | 15 | | Safety and Tolerability of Anakinra in Combination With Riluzol in Amyotrophic Lateral Sclerosis | | Phase 2 | | 20 | | 01/02/2011 | | ND | | N/A | | N/A | | ND | | N/A | | N/A | | No | | Yes | | ND | | N/A | | N/A |
|  | NCT02460679 | |  | |  | | 16 | | Safety and Biomarker Study of EPI-589 in Subjects With ALS | | Phase 2 | | 20 | | 01/11/2015 | | ND | | N/A | | N/A | | ND | | N/A | | N/A | | ND | | ND | | ND | | N/A | | N/A |
|  | NCT03472950 | |  | |  | | 17 | | Safety and Efficacy of Ranolazine for the Treatment of Amyotrophic Lateral Sclerosis | | Phase 2 | | 20 | | 11/06/2018 | | ND | | N/A | | N/A | | ND | | N/A | | N/A | | ND | | ND | | ND | | N/A | | N/A |
|  | NCT01835782 | |  | |  | | 20 | | Determining the Safety of L-serine in ALS | | Phase 1\|Phase 2 | | 20 | | 01/01/2013 | | ND | | N/A | | N/A | | ND | | N/A | | N/A | | ND | | Yes | | ND | | N/A | | N/A |
|  | NCT00324454 | |  | |  | | 21 | | Levetiracetam for Cramps, Spasticity and Neuroprotection in Motor Neuron Disease | | Phase 2 | | 20 | | 01/05/2006 | | ND | | N/A | | N/A | | ND | | N/A | | N/A | | No | | Yes | | ND | | N/A | | N/A |
|  | NCT01020331 | |  | |  | | 22 | | Memantine Therapy in Amyotrophic Lateral Sclerosis | | Phase 2 | | 20 | | 01/06/2005 | | ND | | N/A | | N/A | | ND | | N/A | | N/A | | ND | | ND | | ND | | N/A | | N/A |
|  | NCT02166944 | |  | |  | | 24 | | Tamoxifen Treatment in Patients With Motor Neuron Disease | | Phase 1\|Phase 2 | | 20 | | 01/04/2014 | | ND | | N/A | | N/A | | ND | | N/A | | N/A | | ND | | ND | | ND | | N/A | | N/A |
|  | ND | | 2005-005873-31 | | PMID:19922132 | | 129 | | A randomized, double-blind pilot study vs placebo for the evaluation of efficacy and tolerability of Erytropoietin administered by iv route as add-on treatment in patients affected by ALS | | Phase 2 | | 20 | | 01/08/2005 | | ND | | N/A | | N/A | | ND | | N/A | | N/A | | ND | | ND | | ND | | N/A | | N/A |
|  |  | |  | | PMID:17127563 | | 198 | | Investigation of the therapeutic effects of edaravone, a free radical scavenger, on amyotrophic lateral sclerosis (Phase II study). | | Phase 2 | | 20 | | 1 Nov 2001 | | ND | | N/A | | N/A | | ND | | N/A | | N/A | | ND | | Yes | | ND | | N/A | | N/A |
|  | NCT03272802 | |  | |  | | 19 | | Treatment Effect of Edaravone in Patients With Amyotrophic Lateral Sclerosis (ALS) | | Phase 2\|Phase 3 | | 20 | | 16/03/2017 | | Yes | | ALSAQ-40 | | POM | | ND | | N/A | | N/A | | ND | | Yes | | ND | | N/A | | N/A |
|  |  | |  | | PMID:20839903 | | 222 | | A pilot trial of memantine and riluzole in ALS: Correlation to CSF biomarkers | | ND | | 20 | | ND | | Yes | | SF-36 | | SOM | | ND | | N/A | | N/A | | Yes | | Yes | | ND | | N/A | | N/A |
|  | NCT02469896 | |  | |  | | 25 | | A Trial of Tocilizumab in ALS Subjects | | Phase 2 | | 22 | | 01/11/2015 | | ND | | N/A | | N/A | | ND | | N/A | | N/A | | ND | | Yes | | ND | | N/A | | N/A |
|  | NCT01951924 | |  | |  | | 26 | | LIME Study (LFB IVIg MMN Efficacy Study) | | Phase 3 | | 23 | | 01/12/2013 | | ND | | N/A | | N/A | | ND | | N/A | | N/A | | ND | | Yes | | ND | | N/A | | N/A |
|  | NCT02164253 | |  | |  | | 28 | | Focal Accumulation of Iron in Cerebral Regions in Early ALS (Amyotrophic Lateral Sclerosis) Patients | | Phase 2 | | 23 | | 01/09/2013 | | ND | | N/A | | N/A | | ND | | N/A | | N/A | | No | | Yes | | ND | | N/A | | N/A |
|  |  | |  | | PMID:8777774 | | 183 | | A controlled one-year trial of dextromethorphan in amyotrophic lateral sclerosis. | | ND | | 24 | | ND | | ND | | N/A | | N/A | | ND | | N/A | | N/A | | ND | | ND | | ND | | N/A | | N/A |
|  | NCT03843710 | |  | |  | | 217 | | 31P-MRS Imaging to Assess the Effects of CNM-Au8 on Impaired Neuronal Redox State in Amyotrophic Lateral Sclerosis (REPAIR-ALS) (REPAIR-ALS) | | 2 | | 24 | | 30/04/2020 | | ND | | N/A | | N/A | | ND | | N/A | | N/A | | ND | | Yes | | ND | | N/A | | N/A |
|  | NCT00140452 | |  | | PMID:19922130 \| PMCID:PMC3820489 | | 29 | | Phase II Study Using Thalidomide for the Treatment of ALS | | Phase 2 | | 24 | | 01/02/2005 | | Yes | | ND | | SOM | | ND | | N/A | | N/A | | ND | | Yes | | ND | | N/A | | N/A |
|  |  | |  | | PMID:21321491 | | 221 | | Effects of 3-4 diaminopyridine (DAP) in motor neuron diseases | | Phase II | | 24 | | ND | | Yes | | SIP/ALS-19 | | SOM | | ND | | N/A | | N/A | | ND | | Yes | | ND | | N/A | | N/A |
|  | NCT00886977 | |  | |  | | 31 | | Efficacy and Safety of YAM80 in Amyotrophic Lateral Sclerosis (ALS) | | Phase 2 | | 25 | | 01/04/2009 | | ND | | N/A | | N/A | | ND | | N/A | | N/A | | ND | | Yes | | ND | | N/A | | N/A |
|  |  | |  | | PMID:26191780 | | 165 | | Increased oxidative stress in patients with amyotrophic lateral sclerosis and the effect of edaravone administration. | | ND | | 26 | | ND | | ND | | N/A | | N/A | | ND | | N/A | | N/A | | ND | | Yes | | ND | | N/A | | N/A |
|  | NCT01486849 | |  | |  | | 33 | | Dose Titration Study to Test Safety and Effects of CK-2017357 in Patients With Amyotrophic Lateral Sclerosis (ALS) | | Phase 2 | | 27 | | 01/11/2011 | | ND | | N/A | | N/A | | ND | | N/A | | N/A | | ND | | Yes | | ND | | N/A | | N/A |
|  | NCT00919555 | |  | |  | | 34 | | Combination Therapy in Amyotrophic Lateral Sclerosis (ALS) | | Phase 1\|Phase 2 | | 28 | | 01/06/2008 | | ND | | N/A | | N/A | | ND | | N/A | | N/A | | ND | | ND | | ND | | N/A | | N/A |
|  | NCT03114215 | |  | |  | | 37 | | Effect of MD1003 in Amyotrophic Lateral Sclerosis | | Phase 2 | | 30 | | 29/06/2016 | | ND | | N/A | | N/A | | ND | | N/A | | N/A | | No | | Yes | | ND | | N/A | | N/A |
|  | NCT03186040 | |  | |  | | 40 | | Open-label Clinical Trial of Lacosamide in ALS | | Phase 1\|Phase 2 | | 30 | | 13/07/2017 | | ND | | N/A | | N/A | | ND | | N/A | | N/A | | ND | | Yes | | ND | | N/A | | N/A |
|  | ND | | 2007-004165-17 | | PMID: 25297012 | | 132 | | Efficacy assessment of ceftriaxone therapy in patients with amyotrophic lateral sclerosis | | phase 2 | | 30 | | 25/09/2007 | | ND | | N/A | | N/A | | ND | | N/A | | N/A | | ND | | Yes | | ND | | N/A | | N/A |
|  |  | |  | | PMID:12807386 | | 189 | | A double-blind randomized clinical trial in amyotrophic lateral sclerosis using lamotrigine: effects on CSF glutamate, aspartate, branched-chain amino acid levels and clinical parameters. | | ND | | 30 | | ND | | ND | | N/A | | N/A | | ND | | N/A | | N/A | | ND | | Yes | | ND | | N/A | | N/A |
|  | NCT01884571 | |  | |  | | 42 | | Immunosuppression in Amyotrophic Lateral Sclerosis (ALS) | | Phase 2 | | 31 | | 01/10/2013 | | ND | | N/A | | N/A | | ND | | N/A | | N/A | | ND | | Yes | | ND | | N/A | | N/A |
|  | NCT00877604 | |  | | PMID:25664595 \| PMCID:PMC5024041 | | 43 | | Efficacy and Tolerability of Tauroursodeoxycholic Acid in Amyotrophic Lateral Sclerosis | | Phase 2 | | 34 | | 01/06/2008 | | Yes | | SF-36 | | SOM | | ND | | N/A | | N/A | | No | | Yes | | ND | | N/A | | N/A |
|  | NCT02059759 | |  | |  | | 45 | | Immuno-modulation in Amyotrophic Lateral Sclerosis- a Phase II Study of Safety and Activity of Low Dose Interleukin-2 | | Phase 2 | | 36 | | 01/09/2015 | | ND | | N/A | | N/A | | ND | | N/A | | N/A | | ND | | Yes | | ND | | N/A | | N/A |
|  | NCT01232738 | |  | |  | | 46 | | Trial of Safety and Efficacy of Rasagiline in Patients With Amyotrophic Lateral Sclerosis (ALS) | | Phase 2 | | 36 | | 01/12/2011 | | ND | | N/A | | N/A | | ND | | N/A | | N/A | | ND | | Yes | | ND | | N/A | | N/A |
| JPRN-UMIN000008527 |  | |  | | PMID:26910108 \| PMCID:PMC4765990 | | 169 | | Bromocriptine Mesylate Attenuates Amyotrophic Lateral Sclerosis: A Phase 2a, Randomized, Double-Blind, Placebo-Controlled Research in Japanese Patients. | | 2 | | 36 | | ND | | Yes | | ALSAQ-40 | | POM | | ND | | N/A | | N/A | | ND | | Yes | | ND | | N/A | | N/A |
|  | NCT00753571 | |  | |  | | 49 | | Cistanche Total Glycosides for Amyotrophic Lateral Sclerosis: A Randomized Control Trial (RCT) Study Assessing Clinical Response | | Phase 2 | | 40 | | 01/01/2008 | | ND | | N/A | | N/A | | ND | | N/A | | N/A | | ND | | Yes | | ND | | N/A | | N/A |
|  | NCT00107770 | |  | | PMID:18688762 | | 51 | | Safety Study of Oral Sodium Phenylbutyrate in Subjects With ALS (Amyotrophic Lateral Sclerosis) | | Phase 1\|Phase 2 | | 40 | | 01/04/2005 | | ND | | N/A | | N/A | | ND | | N/A | | N/A | | ND | | Yes | | ND | | N/A | | N/A |
|  | NCT00397423 | |  | |  | | 52 | | G-CSF Treatment for Amyotrophic Lateral Sclerosis: A RCT Study Assessing Clinical Response | | Phase 2 | | 40 | | 01/12/2006 | | ND | | N/A | | N/A | | ND | | N/A | | N/A | | ND | | Yes | | ND | | N/A | | N/A |
|  | ND | | 2011-001329-26 | |  | | 141 | | ErythroPOietin in ALS: a Study of dose-finding and Safety | | Phase 2 | | 40 | | 03/08/2011 | | ND | | N/A | | N/A | | ND | | N/A | | N/A | | No | | Yes | | ND | | N/A | | N/A |
|  | ND | | 2012-002099-15 | |  | | 142 | | A randomised, double blind, placebo controlled trial to evaluate the safety and efficacy of Apovir for treatment of patients with Amyotrophic lateral sclerosis | | Phase 2 | | 40 | | 15/09/2014 | | ND | | N/A | | N/A | | ND | | N/A | | N/A | | No | | Yes | | ND | | N/A | | N/A |
|  | NCT03204500 | |  | |  | | 54 | | Dual Treatment With Lithium and Valproate in ALS. | | Phase 2 | | 40 | | 01/05/2016 | | Yes | | ALSAQ-5 | | SOM | | ND | | N/A | | N/A | | ND | | Yes | | ND | | N/A | | N/A |
|  | NCT00635960 | |  | |  | | 53 | | Growth Hormone in Amyotrophic Lateral Sclerosis | | Phase 2 | | 40 | | 01/03/2007 | | Yes | | SF-36 | | SOM | | ND | | N/A | | N/A | | ND | | ND | | ND | | N/A | | N/A |
|  | NCT00036413 | |  | |  | | 59 | | A 12-week, Multicenter, Safety and Dose-ranging Study of 3 Oral Doses of TCH346 in Patients With Amyotrophic Lateral Sclerosis | | Phase 2 | | 44 | | 01/01/2002 | | ND | | N/A | | N/A | | ND | | N/A | | N/A | | ND | | ND | | ND | | N/A | | N/A |
|  | NCT01378676 | |  | | PMID:23952636 | | 62 | | A Study to Evaluate the Effects of Multiple Doses of CK-2017357 in Patients With Amyotrophic Lateral Sclerosis (ALS) | | Phase 2 | | 49 | | 01/06/2011 | | ND | | N/A | | N/A | | ND | | N/A | | N/A | | ND | | Yes | | ND | | N/A | | N/A |
|  | NCT03580616 | |  | |  | | 64 | | Tolerability and Efficacy of L-Serine in Patients With Amyotrophic Lateral Sclerosis (ALS) | | Phase 2 | | 50 | | 24/10/2018 | | ND | | N/A | | N/A | | ND | | N/A | | N/A | | Yes | | ND | | ND | | N/A | | N/A |
|  | NCT03693781 | |  | |  | | 65 | | Colchicine for Amyotrophic Lateral Sclerosis | | Phase 2 | | 54 | | 30/01/2019 | | Yes | | ALSAQ-40 | | SOM | | ND | | N/A | | N/A | | ND | | Yes | | ND | | N/A | | N/A |
|  |  | |  | | PMID:8681311 | | 159 | | The pharmacokinetics of subcutaneously administered recombinant human ciliary neurotrophic factor (rHCNTF) in patients with amyotrophic lateral sclerosis: relation to parameters of the acute-phase response. The ALS CNTF Treatment Study (ACTS) Phase I-II Study Group. | | Phase 1/2 | | 57 | | ND | | ND | | N/A | | N/A | | ND | | N/A | | N/A | | ND | | ND | | ND | | N/A | | N/A |
| JPRN-UMIN000006423 |  | |  | | PMID:25960085 | | 170 | | A single blind randomized controlled clinical trial of mexiletine in amyotrophic lateral sclerosis: Efficacy and safety of sodium channel blocker phase II trial. | | 2 | | 60 | | ND | | ND | | N/A | | N/A | | ND | | N/A | | N/A | | ND | | ND | | ND | | N/A | | N/A |
|  | NCT03359538 | |  | | PMID:29901635 \| PMCID:PMC6024184 | | 71 | | Rapamycin Treatment for ALS | | Phase 2 | | 63 | | 19/09/2017 | | Yes | | ALSAQ-40 | | SOM | | ND | | N/A | | N/A | | ND | | Yes | | ND | | N/A | | N/A |
|  |  | |  | | PMID:8304845 | | 177 | | Immunosuppressive treatment of motor neuron syndromes. Attempts to distinguish a treatable disorder. | | Phase I/II | | 65 | | 01/01/1989 | | ND | | N/A | | N/A | | ND | | N/A | | N/A | | ND | | ND | | ND | | N/A | | N/A |
|  | NCT01089010 | |  | | PMID:23952600\|PMID:22591195 | | 74 | | A Study of CK-2017357 in Patients With Amyotrophic Lateral Sclerosis (ALS) | | Phase 2 | | 67 | | 01/03/2010 | | ND | | N/A | | N/A | | ND | | N/A | | N/A | | ND | | Yes | | ND | | N/A | | N/A |
|  |  | |  | | PMID:11732275 | | 191 | | [The effect of selegiline and vitamin E in the treatment of ALS: an open randomized clinical trials]. | | ND | | 67 | | ND | | ND | | N/A | | N/A | | ND | | N/A | | N/A | | ND | | Yes | | ND | | N/A | | N/A |
|  |  | |  | | PMID:8622731 | | 184 | | A clinical trial of verapamil in amyotrophic lateral sclerosis. | | ND | | 72 | | 01/09/1991 | | ND | | N/A | | N/A | | ND | | N/A | | N/A | | ND | | Yes | | ND | | N/A | | N/A |
|  | NCT00244244 | |  | | PMID:18551622 | | 77 | | A Multicenter, Dose Ranging Safety and Pharmacokinetics Study of Arimoclomol in ALS | | Phase 2 | | 80 | | 01/10/2005 | | ND | | N/A | | N/A | | ND | | N/A | | N/A | | ND | | ND | | ND | | N/A | | N/A |
|  | ND | | 2005-005152-40 | |  | | 128 | | MULTICENTER, DOUBLE-BLIND, RANDOMIZED, PLACEBO-CONTROLLED, TRIAL ON ALPHA-LIPOIC ACID FOR THE TREATMENT OF AMYOTROPHIC LATERAL SCLEROSIS ALALS | | Phase 2 | | 84 | | 19/04/2006 | | ND | | N/A | | N/A | | ND | | N/A | | N/A | | ND | | Yes | | ND | | N/A | | N/A |
|  |  | |  | | PMID:8664560 | | 185 | | Controlled trial of nimodipine in amyotrophic lateral sclerosis. | | ND | | 87 | | ND | | ND | | N/A | | N/A | | ND | | N/A | | N/A | | ND | | Yes | | ND | | N/A | | N/A |
|  | NCT00790582 | |  | | PMID:21813790 \| PMCID:PMC3171956 | | 85 | | A Multi-Center Controlled Screening Trial of Safety and Efficacy of Lithium Carbonate in Subjects With Amyotrophic Lateral Sclerosis (ALS) | | Phase 2 | | 100 | | 01/05/2008 | | ND | | N/A | | N/A | | ND | | N/A | | N/A | | ND | | Yes | | ND | | N/A | | N/A |
| RBR-2n5mtq |  | |  | |  | | 220 | | Clinical and electromyographic follow-up of patients taking riluzole and lithium carbonate - A randomized controlled trial | | Phase II/III | | 100 | | 12/2/2014 | | ND | | N/A | | N/A | | ND | | N/A | | N/A | | ND | | Yes | | ND | | N/A | | N/A |
|  | NCT00647296 | |  | | PMID:22985432\|PMID:22101764 | | 87 | | Safety and Tolerability Study of KNS-760704 in Amyotrophic Lateral Sclerosis (ALS) | | Phase 2 | | 102 | | 01/03/2008 | | ND | | N/A | | N/A | | ND | | N/A | | N/A | | ND | | ND | | ND | | N/A | | N/A |
|  |  | |  | | PMID:15534251 | | 188 | | A clinical trial of creatine in ALS. | | ND | | 104 | | 05/09/2000 | | ND | | N/A | | N/A | | ND | | N/A | | N/A | | ND | | Yes | | ND | | N/A | | N/A |
|  | NCT00069186 | |  | |  | | 88 | | Study of Creatine Monohydrate in Patients With Amyotrophic Lateral Sclerosis | | Phase 3 | | 107 | | 01/06/2003 | | Yes | | ALSFRS-R and SF-12 | | SOM | | ND | | N/A | | N/A | | ND | | ND | | ND | | N/A | | N/A |
|  | NCT00070993 | |  | | PMID:18608103 \| PMCID:PMC2631354 | | 89 | | Creatine for the Treatment of Amyotrophic Lateral Sclerosis | | Phase 2 | | 110 | | 01/12/2002 | | Yes | | SF-12 | | SOM | | ND | | N/A | | N/A | | ND | | ND | | ND | | N/A | | N/A |
|  |  | |  | | PMID:8195821 | | 178 | | An open-randomized clinical trial of selegiline in amyotrophic lateral sclerosis. | | ND | | 111 | | ND | | ND | | N/A | | N/A | | ND | | N/A | | N/A | | No | | Yes | | ND | | N/A | | N/A |
|  | NCT03548311 | |  | |  | | 91 | | Clinical Trial of Ultra-high Dose Methylcobalamin for ALS | | Phase 3 | | 128 | | 01/11/2017 | | Yes | | ALSAQ-40 | | SOM | | ND | | N/A | | N/A | | No | | Yes | | ND | | N/A | | N/A |
|  |  | |  | | PMID:11294919 | | 194 | | Phase III randomized trial of gabapentin in patients with amyotrophic lateral sclerosis. | | ND | | 128 | | ND | | Yes | | SF-12 | | SOM | | ND | | N/A | | N/A | | ND | | Yes | | ND | | N/A | | N/A |
|  |  | |  | | PMID:9443715 | | 196 | | Selegiline is ineffective in a collaborative double-blind, placebo-controlled trial for treatment of amyotrophic lateral sclerosis. | | ND | | 133 | | ND | | ND | | N/A | | N/A | | ND | | N/A | | N/A | | ND | | Yes | | ND | | N/A | | N/A |
|  | NCT02794857 | |  | |  | | 95 | | Safety and Efficacy Study of NP001 in Patients With Amyotrophic Lateral Sclerosis (ALS) and Systemic Inflammation | | Phase 2 | | 138 | | 29/08/2016 | | ND | | N/A | | N/A | | ND | | N/A | | N/A | | ND | | ND | | ND | | N/A | | N/A |
|  | ND | | 2007-002117-39 | |  | | 130 | | Memantine for functional disability in ALS | | Phase 2 | | 140 | | 31/01/2008 | | ND | | N/A | | N/A | | ND | | N/A | | N/A | | No | | Yes | | ND | | N/A | | N/A |
|  | NCT03456882 | |  | |  | | 97 | | The Effect of RNS60 on ALS Biomarkers | | Phase 2 | | 142 | | 18/11/2016 | | Yes | | ALSAQ-40 | | SOM | | ND | | N/A | | N/A | | ND | | Yes | | ND | | N/A | | N/A |
|  | NCT03068754 | |  | |  | | 98 | | Study of ActharÂ® Gel (Acthar) for Amyotrophic Lateral Sclerosis (ALS) | | Phase 2\|Phase 3 | | 143 | | 22/06/2017 | | ND | | N/A | | N/A | | ND | | N/A | | N/A | | ND | | ND | | ND | | N/A | | N/A |
|  | NCT02623699 | |  | |  | | 99 | | An Efficacy, Safety, Tolerability, Pharmacokinetics and Pharmacodynamics Study of BIIB067 in Adults With Inherited Amyotrophic Lateral Sclerosis (ALS) | | Phase 3 | | 144 | | 20/01/2016 | | Yes | | ALSAQ-5 | | SOM | | ND | | N/A | | N/A | | ND | | ND | | ND | | N/A | | N/A |
|  |  | |  | | PMID:8960715 | | 181 | | Placebo-controlled trial of gabapentin in patients with amyotrophic lateral sclerosis. WALS Study Group. Western Amyotrophic Lateral Sclerosis Study Group. | | Phase II | | 152 | | ND | | ND | | N/A | | N/A | | ND | | N/A | | N/A | | ND | | Yes | | ND | | N/A | | N/A |
|  | ND | | 2009-016066-91 | | PMID:25595151 \|PMID:25886781 | | 139 | | SAFETY AND EFFICACY OF ERYTHROPOIETIN IN AMYOTROPHIC LATERAL SCLEROSIS: A RANDOMIZED, PLACEBO-CONTROLLED CLINICAL TRIAL | | Phase 3 | | 160 | | 10/03/2010 | | ND | | N/A | | N/A | | ND | | N/A | | N/A | | ND | | Yes | | ND | | N/A | | N/A |
|  |  | |  | | PMID:15517433 | | 187 | | High dose vitamin E therapy in amyotrophic lateral sclerosis as add-on therapy to riluzole: results of a placebo-controlled double-blind study. | | ND | | 160 | | 01/12/1998 | | Yes | | SIP | | SOM | | ND | | N/A | | N/A | | ND | | ND | | ND | | N/A | | N/A |
|  | NCT00136110 | |  | | PMID:19743466 | | 100 | | Trial of Sodium Valproate in Amyotrophic Lateral Sclerosis | | Phase 3 | | 165 | | 01/04/2005 | | ND | | N/A | | N/A | | ND | | N/A | | N/A | | ND | | Yes | | ND | | N/A | | N/A |
|  | NCT00424463 | |  | |  | | 101 | | Expanded Controlled Study of Safety and Efficacy of MCI-186 in Patients With Amyotrophic Lateral Sclerosis (ALS) | | Phase 3 | | 181 | | 01/01/2007 | | ND | | N/A | | N/A | | ND | | N/A | | N/A | | No | | ND | | ND | | N/A | | N/A |
|  | NCT03491462 | |  | |  | | 105 | | Arimoclomol in Amyotropic Lateral Sclerosis | | Phase 3 | | 231 | | 31/07/2018 | | ND | | N/A | | N/A | | ND | | N/A | | N/A | | ND | | ND | | ND | | N/A | | N/A |
|  |  | |  | | PMID:9409357 | | 176 | | Effect of recombinant human insulin-like growth factor-I on progression of ALS. A placebo-controlled study. The North America ALS/IGF-I Study Group. | | ND | | 266 | | ND | | Yes | | SIP | | SOM | | ND | | N/A | | N/A | | No | | ND | | ND | | N/A | | N/A |
|  |  | |  | | PMID:16802291 | | 199 | | Trial of celecoxib in amyotrophic lateral sclerosis. | | ND | | 300 | | ND | | ND | | N/A | | N/A | | ND | | N/A | | N/A | | ND | | Yes | | ND | | N/A | | N/A |
|  | NCT00326625 | |  | | PMID:19922128 | | 111 | | Clinical Trial of Glatiramer Acetate in Amyotrophic Lateral Sclerosis (ALS) | | Phase 2 | | 366 | | 31/07/2006 | | ND | | N/A | | N/A | | ND | | N/A | | N/A | | ND | | Yes | | ND | | N/A | | N/A |
|  | NCT02588677 | |  | |  | | 113 | | Masitinib in Combination With Riluzole for the Treatment of Patients Suffering From Amyotrophic Lateral Sclerosis (ALS) | | Phase 2\|Phase 3 | | 394 | | 01/04/2013 | | ND | | N/A | | N/A | | ND | | N/A | | N/A | | ND | | ND | | ND | | N/A | | N/A |
|  | NCT00403104 | |  | |  | | 115 | | Placebo Controlled Study of ONO2506PO in the Presence of Riluzole in Patients With Amyotrophic Lateral Sclerosis (ALS) | | Phase 2 | | 420 | | 01/11/2006 | | Yes | | ND | | SOM | | ND | | N/A | | N/A | | ND | | ND | | ND | | N/A | | N/A |
|  | NCT00349622 | |  | | PMID:27385750\|PMID:26824413\|PMID:25297012\|PMID:24771634\|PMID:23613806 | | 121 | | Clinical Trial Ceftriaxone in Subjects With ALS | | Phase 3 | | 513 | | 01/07/2006 | | Yes | | ALSQOL-R | | SOM | | ND | | N/A | | N/A | | ND | | ND | | ND | | N/A | | N/A |
|  | NCT00696332 | |  | |  | | 123 | | Talampanel for Amyotrophic Lateral Sclerosis (ALS) | | Phase 2 | | 559 | | 01/09/2008 | | ND | | N/A | | N/A | | ND | | N/A | | N/A | | ND | | ND | | ND | | N/A | | N/A |
|  |  | |  | | PMID:10227630 | | 160 | | A controlled trial of recombinant methionyl human BDNF in ALS: The BDNF Study Group (Phase III). | | Phase 3 | | 1135 | | July 1995 | | Yes | | SIP | | SOM | | ND | | N/A | | N/A | | ND | | Yes | | ND | | N/A | | N/A |
|  |  | |  | | PMID:12939417 | | 208 | | A randomized, placebo-controlled trial of topiramate in amyotrophic lateral sclerosis. | | ND | | 0 | | ND | | ND | | N/A | | N/A | | ND | | N/A | | N/A | | ND | | Yes | | ND | | N/A | | N/A |
|  |  | |  | | PMID:14506939 | | 209 | | Reduction of oxidative stress in amyotrophic lateral sclerosis following pramipexole treatment. | | ND | | 0 | | ND | | ND | | N/A | | N/A | | ND | | N/A | | N/A | | No | | Yes | | ND | | N/A | | N/A |
|  |  | |  | | PMID:8909433 | | 162 | | A controlled trial of amino acid therapy in amyotrophic lateral sclerosis: I. Clinical, functional, and maximum isometric torque data. | | ND | | 95 | | ND | | ND | | N/A | | N/A | | ND | | N/A | | N/A | | ND | | Yes | | ND | | N/A | | N/A |
| IRCT2015062411424N3 |  | |  | | PMID:29352425 \| PMCID:PMC5935637 | | 167 | | Safety and Efficacy of Nanocurcumin as Add-On Therapy to Riluzole in Patients With Amyotrophic Lateral Sclerosis: A Pilot Randomized Clinical Trial. | | ND | | 54 | | ND | | ND | | N/A | | N/A | | ND | | N/A | | N/A | | ND | | ND | | ND | | N/A | | N/A |
|  |  | |  | | PMID:19961264 | | 173 | | A phase II trial of talampanel in subjects with amyotrophic lateral sclerosis. | | phase 2 | | 59 | | ND | | ND | | N/A | | N/A | | ND | | N/A | | N/A | | ND | | ND | | ND | | N/A | | N/A |
|  |  | |  | | PMID:7931231 | | 179 | | Double blind cross over trial with deprenyl in amyotrophic lateral sclerosis. | | ND | | 10 | | ND | | ND | | N/A | | N/A | | ND | | N/A | | N/A | | No | | ND | | ND | | N/A | | N/A |
|  |  | |  | | PMID:8628460 | | 157 | | A double-blind placebo-controlled clinical trial of subcutaneous recombinant human ciliary neurotrophic factor (rHCNTF) in amyotrophic lateral sclerosis. ALS CNTF Treatment Study Group. | | ND | | 730 | | ND | | ND | | N/A | | N/A | | ND | | N/A | | N/A | | ND | | ND | | ND | | N/A | | N/A |
|  | ND | | 2008-006224-61 | |  | | 135 | | Multicenter, randomized, controlled clinical trial to evaluate the efficacy of lithium carbonate treatment in patients with Amyotrophic Lateral Sclerosis | | ND | | 120 | | 12/06/2009 | | ND | | N/A | | N/A | | ND | | N/A | | N/A | | ND | | Yes | | ND | | N/A | | N/A |
| NCT00005674 | NCT00005674 | |  | |  | | 155 | | Clinical Trial of Creatine in Amyotrophic Lateral Sclerosis [ALS] | | ND | | 114 | | ND | | ND | | N/A | | N/A | | ND | | N/A | | N/A | | ND | | Yes | | ND | | N/A | | N/A |
|  |  | |  | | PMID:8909453 | | 156 | | Toxicity and tolerability of recombinant human ciliary neurotrophic factor in patients with amyotrophic lateral sclerosis. | | ND | | 72 | | ND | | ND | | N/A | | N/A | | ND | | N/A | | N/A | | ND | | Yes | | ND | | N/A | | N/A |
|  |  | |  | | PMID:8967757 | | 158 | | A placebo-controlled trial of recombinant human ciliary neurotrophic (rhCNTF) factor in amyotrophic lateral sclerosis. rhCNTF ALS Study Group. | | ND | | 570 | | ND | | ND | | N/A | | N/A | | ND | | N/A | | N/A | | ND | | ND | | ND | | N/A | | N/A |
|  |  | |  | | PMID:8302340 | | 161 | | A controlled trial of riluzole in amyotrophic lateral sclerosis. ALS/Riluzole Study Group. | | ND | | 78 | | ND | | ND | | N/A | | N/A | | ND | | N/A | | N/A | | No | | Yes | | ND | | N/A | | N/A |
|  |  | |  | | PMID:8145910 | | 163 | | Intravenous immunoglobulin treatment in patients with motor neuron syndromes associated with anti-GM1 antibodies: a double-blind, placebo-controlled study. | | ND | | 12 | | ND | | ND | | N/A | | N/A | | ND | | N/A | | N/A | | ND | | ND | | ND | | N/A | | N/A |
|  |  | |  | | PMID:10668716 | | 195 | | A randomized controlled trial of recombinant interferon beta-1a in ALS. Italian Amyotrophic Lateral Sclerosis Study Group. | | ND | | 37 | | ND | | ND | | N/A | | N/A | | ND | | N/A | | N/A | | No | | Yes | | ND | | N/A | | N/A |
|  |  | |  | | PMID:17127564 | | 197 | | A pilot trial with clenbuterol in amyotrophic lateral sclerosis. | | ND | | 16 | | ND | | ND | | N/A | | N/A | | ND | | N/A | | N/A | | ND | | Yes | | ND | | N/A | | N/A |
|  |  | |  | | PMID:16606934 | | 200 | | Randomized controlled phase II trial of glatiramer acetate in ALS. | | ND | | 30 | | ND | | ND | | N/A | | N/A | | ND | | N/A | | N/A | | Yes | | Yes | | ND | | N/A | | N/A |
|  |  | |  | | PMID:16401852 | | 201 | | Pentoxifylline in ALS: a double-blind, randomized, multicenter, placebo-controlled trial. | | ND | | 400 | | ND | | ND | | N/A | | N/A | | ND | | N/A | | N/A | | ND | | Yes | | ND | | N/A | | N/A |
|  |  | |  | | PMID:16197815 | | 202 | | Beneficial effects of intrathecal IGF-1 administration in patients with amyotrophic lateral sclerosis. | | ND | | 9 | | July 2000 | | ND | | N/A | | N/A | | ND | | N/A | | N/A | | ND | | Yes | | ND | | N/A | | N/A |
|  |  | |  | | PMID:16193258 | | 203 | | Minocycline in amyotrophic lateral sclerosis: a pilot study. | | ND | | 20 | | ND | | ND | | N/A | | N/A | | ND | | N/A | | N/A | | ND | | ND | | ND | | N/A | | N/A |
|  |  | |  | | PMID:11677005 | | 210 | | Effects of creatine supplementation on exercise performance and muscular strength in amyotrophic lateral sclerosis: preliminary results. | | ND | | 28 | | ND | | ND | | N/A | | N/A | | ND | | N/A | | N/A | | ND | | Yes | | ND | | N/A | | N/A |
|  |  | |  | | PMID:10935831 | | 211 | | Reduced glutathione in amyotrophic lateral sclerosis: an open, crossover, randomized trial. | | ND | | 32 | | ND | | ND | | N/A | | N/A | | ND | | N/A | | N/A | | ND | | ND | | ND | | N/A | | N/A |
|  |  | |  | | PMID:9851651 | | 212 | | The natural history and the effects of gabapentin in amyotrophic lateral sclerosis. | | ND | | 110 | | ND | | ND | | N/A | | N/A | | ND | | N/A | | N/A | | ND | | Yes | | ND | | N/A | | N/A |
|  |  | |  | | PMID:9864711 | | 214 | | [Assessment of the efficacy of treatment with pimozide in patients with amyotrophic lateral sclerosis. Introductory notes]. | | ND | | 44 | | ND | | ND | | N/A | | N/A | | ND | | N/A | | N/A | | ND | | ND | | ND | | N/A | | N/A |
|  |  | |  | | PMID:9262126 | | 215 | | A clinical trial of dextromethorphan in amyotrophic lateral sclerosis. | | ND | | 45 | | ND | | ND | | N/A | | N/A | | ND | | N/A | | N/A | | ND | | ND | | ND | | N/A | | N/A |
|  |  | |  | | PMID:7807153 | | 216 | | Treatment of ALS with high dose pulse cyclophosphamide. | | ND | | 18 | | ND | | ND | | N/A | | N/A | | ND | | N/A | | N/A | | ND | | ND | | ND | | N/A | | N/A |
|  | ND | | 2017-001983-39 | |  | | 145 | | A placebo-controlled double blind randomized trial to investigate the efficacy and safety of the combination of Penicillin G / Hydrocortisone treatment in ALS patients (PHALS) | | ND | | 12 | | 28/09/2017 | | Yes | | EQ-5D-5L | | SOM | | ND | | N/A | | N/A | | No | | ND | | ND | | N/A | | N/A |
|  | ND | | 2008-005106-38 | |  | | 134 | | Double Blind Randomized Clinical Trial Of Lithium Carbonate As Association Therapy In ALS(Phase II) | | Phase 2 | | 280 | | 14/05/2009 | | Yes | | ND | | SOM | | ND | | N/A | | N/A | | No | | Yes | | ND | | N/A | | N/A |
|  |  | |  | | PMID:15824372 | | 205 | | A pilot, double-blind, placebo-controlled trial of indinavir in patients with ALS. | | ND | | 46 | | ND | | Yes | | SF-36 | | SOM | | ND | | N/A | | N/A | | No | | Yes | | ND | | N/A | | N/A |
|  |  | |  | | PMID:15204012 | | 206 | | Efficacy and safety of xaliproden in amyotrophic lateral sclerosis: results of two phase III trials. | | ND | | 1210 | | ND | | Yes | | SIP | | SOM | | ND | | N/A | | N/A | | ND | | Yes | | ND | | N/A | | N/A |
|  |  | |  | | PMID:9710040 | | 213 | | A placebo-controlled trial of insulin-like growth factor-I in amyotrophic lateral sclerosis. European ALS/IGF-I Study Group. | | ND | | 59 | | ND | | Yes | | SIP | | SOM | | ND | | N/A | | N/A | | ND | | ND | | ND | | N/A | | N/A |
| NTR1448 |  | |  | | PMID:22378918 | | 219 | | Lithium lacks effect on survival in amyotrophic lateral sclerosis: a phase IIb randomised sequential trial | | ND | | 113 | | 1/11/2008 | | ND | | N/A | | N/A | | ND | | N/A | | N/A | | ND | | Yes | | ND | | N/A | | N/A |
|  | NCT03474263 | |  | |  | | 1 | | IC14 for Rapidly Progressive Amyotrophic Lateral Sclerosis (ALS) | | Phase 2 | | 0 | | 01/09/2019 | | ND | | N/A | | N/A | | Yes | | ND | | EC | | ND | | Yes | | ND | | N/A | | N/A |
|  | NCT00561366 | |  | |  | | 3 | | Safety and Efficacy of Arimoclomol in Volunteers With ALS | | Phase 2 | | 0 | | 20/11/2007 | | Yes | | ND | | SOM | | Yes | | ND | | EC | | ND | | ND | | ND | | N/A | | N/A |
|  | NCT02039401 | |  | | PMID:28166654 | | 180 | | Open label study to assess the safety of VM202 in subjects with amyotrophic lateral sclerosis. | | phase I/II | | 18 | | 01/01/2014 | | ND | | N/A | | N/A | | Yes | | ND | | EC | | ND | | Yes | | ND | | N/A | | N/A |
|  | NCT02463825 | |  | |  | | 30 | | A Registry-Based Clinical Trial of Pimozide in Patients With Neuromuscular Junction Transmission Dysfunction Due to ALS | | Phase 2 | | 25 | | 01/04/2015 | | ND | | N/A | | N/A | | Yes | | ND | | EC | | ND | | ND | | ND | | N/A | | N/A |
|  | NCT00706147 | |  | | PMID:29367439 \| PMCID:PMC5818014 | | 47 | | Phase II/III Randomized, Placebo-controlled Trial of Arimoclomol in SOD1 Positive Familial Amyotrophic Lateral Sclerosis | | Phase 2\|Phase 3 | | 38 | | 01/01/2009 | | ND | | N/A | | N/A | | Yes | | ND | | EC | | ND | | Yes | | ND | | N/A | | N/A |
|  | NCT00231140 | |  | |  | | 50 | | Pilot-Study of Thalidomide in Amyotrophic Lateral Sclerosis (ALS) | | Phase 2 | | 40 | | 01/12/2005 | | ND | | N/A | | N/A | | Yes | | ND | | EC | | No | | Yes | | ND | | N/A | | N/A |
|  | NCT00818389 | |  | | PMID:20363190 \| PMCID:PMC3071495 | | 80 | | Study to Investigate the Safety and Efficacy of Lithium in Volunteers With Amyotrophic Lateral Sclerosis (ALS) | | Phase 2\|Phase 3 | | 84 | | 01/01/2009 | | ND | | N/A | | N/A | | Yes | | ND | | EC | | ND | | Yes | | ND | | N/A | | N/A |
|  | ND | | 2014-002228-28 | |  | | 143 | | A double blind, placebo controlled, parallel groups, multicenter study on filgrastim in amyotrophic lateral sclerosis | | Phase 2 | | 90 | | 01/09/2014 | | ND | | N/A | | N/A | | Yes | | ND | | EC | | ND | | Yes | | ND | | N/A | | N/A |
|  | NCT00214110 | |  | |  | | 86 | | Tamoxifen Therapy in Amyotrophic Lateral Sclerosis [ALS] | | Phase 2 | | 100 | | 01/01/2001 | | ND | | N/A | | N/A | | Yes | | ND | | EC | | ND | | ND | | ND | | N/A | | N/A |
|  |  | |  | | PMID:7763202 | | 186 | | Randomized, double-blind, controlled trial of acetylcysteine in amyotrophic lateral sclerosis. | | ND | | 110 | | ND | | ND | | N/A | | N/A | | Yes | | ND | | EC | | No | | Yes | | ND | | N/A | | N/A |
|  | NCT01281631 | |  | |  | | 93 | | A Study of NP001 in Subjects With Amyotrophic Lateral Sclerosis (ALS) | | Phase 2 | | 136 | | 01/02/2011 | | ND | | N/A | | N/A | | Yes | | ND | | EC | | ND | | ND | | ND | | N/A | | N/A |
|  | NCT00243932 | |  | | PMID:23452274\|PMID:19743457 | | 102 | | Clinical Trial of High Dose CoQ10 in ALS | | Phase 2 | | 185 | | 01/04/2005 | | ND | | N/A | | N/A | | Yes | | ND | | EC | | ND | | Yes | | ND | | N/A | | N/A |
|  | NCT00330681 | |  | | PMID:28872919\|PMID:28872917\|PMID:25286015 | | 103 | | Efficacy and Safety Study of MCI-186 for Treatment of Amyotrophic Lateral Sclerosis (ALS) | | Phase 3 | | 206 | | 01/05/2006 | | Yes | | ALSAQ-40 | | SOM | | Yes | | ND | | EC | | No | | ND | | ND | | N/A | | N/A |
|  | NCT00690118 | |  | | PMID:26984187\|PMID:22715372 | | 104 | | Study of Pioglitazone in Patients With Amyotrophic Lateral Sclerosis | | Phase 2 | | 219 | | 01/05/2008 | | ND | | N/A | | N/A | | Yes | | ND | | EC | | No | | ND | | ND | | N/A | | N/A |
|  | NCT03039673 | |  | |  | | 108 | | MIROCALS: Modifying Immune Response and OutComes in ALS | | Phase 2 | | 304 | | 19/06/2017 | | ND | | N/A | | N/A | | Yes | | ND | | EC | | ND | | ND | | ND | | N/A | | N/A |
|  | NCT01753076 | |  | | PMID:28139349 | | 109 | | Study of Ozanezumab (GSK1223249) Versus Placebo in the Treatment of Amyotrophic Lateral Sclerosis | | Phase 2 | | 304 | | 20/12/2012 | | Yes | | EQ-5D-5L | | SOM | | Yes | | ND | | EC | | No | | ND | | ND | | N/A | | N/A |
|  | NCT03160898 | |  | |  | | 118 | | A Study to Evaluate Efficacy, Safety and Tolerability of CK-2127107 in Patients With Amyotrophic Lateral Sclerosis (ALS) | | Phase 2 | | 458 | | 24/07/2017 | | ND | | N/A | | N/A | | Yes | | ND | | EC | | ND | | ND | | ND | | N/A | | N/A |
|  | NCT03792490 | |  | |  | | 90 | | Inhibition of Rho Kinase (ROCK) With Fasudil as Disease-modifying Treatment for ALS | | Phase 2 | | 120 | | 20/02/2019 | | Yes | | ALSAQ-5 | | SOM | | Yes | | History | | EC | | No | | ND | | Yes | | ND | | EC |
|  | NCT03800524 | |  | |  | | 116 | | Safety and Efficacy of TUDCA as add-on Treatment in Patients Affected by ALS | | Phase 3 | | 440 | | 22/02/2019 | | Yes | | ALSAQ-40 | | SOM | | Yes | | History | | EC | | No | | ND | | Yes | | ND | | EC |
|  | NCT03705390 | |  | |  | | 11 | | A Safety and Tolerability Study of ILB in Patients With Amyothrophic Lateral Sclerosis (ALS) | | Phase 2 | | 15 | | 29/03/2019 | | Yes | | ALSAQ-40 | | SOM | | Yes | | Investigator Judgement | | EC | | No | | ND | | Yes | | ND | | EC |
|  | NCT03377309 | |  | |  | | 18 | | Safety and Tolerability of Perampanel in Amyotrophic Lateral Sclerosis Patients | | Phase 2 | | 20 | | 01/12/2017 | | ND | | N/A | | N/A | | Yes | | Investigator Judgement | | EC | | No | | ND | | Yes | | ND | | EC |
|  | NCT02988297 | |  | |  | | 96 | | Nebulized RNS60 for the Treatment of Amyotrophic Lateral Sclerosis | | Phase 2 | | 140 | | 01/02/2020 | | Yes | | ALSAQ-40 | | SOM | | Yes | | Investigator Judgement | | EC | | No | | Yes | | Yes | | ND | | EC |
|  | ND | | 2017-002754-36 | |  | | 146 | | Effects of oral Levosimendan (ODM-109) on respiratory function in patients with ALS | | ND | | 0 | | 04/05/2018 | | ND | | N/A | | N/A | | Yes | | Investigator Judgement | | EC | | No | | Yes | | Yes | | ND | | EC |
|  | NCT03883581 | |  | |  | | 55 | | Impact of Nuedexta on Bulbar Physiology and Function in ALS | | Phase 1\|Phase 2 | | 40 | | 25/07/2019 | | ND | | N/A | | N/A | | ND | | N/A | | N/A | | No | | ND | | Yes | | ND | | EC |
|  | NCT01825551 | |  | |  | | 48 | | The Effect of GCSF in the Treatment of ALS Patients | | Phase 2\|Phase 3 | | 40 | | 01/11/2012 | | Yes | | ALSAQ-40 | | SOM | | ND | | N/A | | N/A | | ND | | ND | | Yes | | ND | | EC |
|  | NCT03019419 | |  | |  | | 68 | | Perampanel for Sporadic Amyotrophic Lateral Sclerosis (ALS) | | Phase 2 | | 60 | | 24/04/2017 | | ND | | N/A | | N/A | | ND | | N/A | | N/A | | ND | | Yes | | Yes | | ND | | EC |
|  | NCT01786603 | |  | | PMID:30192007 \| PMCID:PMC6545236 | | 78 | | Rasagiline in Subjects With Amyotrophic Lateral Sclerosis (ALS) | | Phase 2 | | 80 | | 01/09/2012 | | Yes | | ALSQOL-R | | SOM | | ND | | N/A | | N/A | | ND | | Yes | | Yes | | ND | | EC |
|  | ND | | 2004-004158-23 | | PMID:23421600 | | 127 | | DOUBLE-BLIND PLACEBO-CONTROLLED TRIAL ON THE USE OF ACETYL-L-CARNITINE FOR THE TREATMENT OF AMYOTROPHIC LATERAL SCLEROSIS (ALS) | | Phase 2 | | 80 | | 15/04/2005 | | Yes | | McGill | | SOM | | ND | | N/A | | N/A | | ND | | Yes | | Yes | | ND | | EC |
|  | NCT00355576 | |  | | PMID:18608093 \| PMCID:PMC4354803 | | 81 | | Combination Therapy Selection Trial in Amyotrophic Lateral Sclerosis | | Phase 2 | | 86 | | 01/07/2006 | | Yes | | McGill | | SOM | | ND | | N/A | | N/A | | ND | | Yes | | Yes | | ND | | EC |
|  | ND | | 2008-001094-15 | | PMID:20702794 | | 133 | | SINGLE-BLIND, RANDOMIZED, PARALLEL GROUP, DOSE-FINDING TRIAL ON LITHIUM FOR THE TREATMENT OF AMYOTROPHIC LATERAL SCLEROSIS (ALS) | | Phase 2 | | 150 | | 10/03/2008 | | Yes | | McGill | | SOM | | ND | | N/A | | N/A | | ND | | Yes | | Yes | | ND | | EC |
|  | NCT00444613 | |  | |  | | 112 | | A Study in Patients With Amyotrophic Lateral Sclerosis (ALS) | | Phase 2\|Phase 3 | | 373 | | 01/04/2007 | | ND | | N/A | | N/A | | ND | | N/A | | N/A | | ND | | Yes | | Yes | | ND | | EC |
|  | NCT00047723 | |  | | PMID: 17980667 | | 114 | | Minocycline to Treat Amyotrophic Lateral Sclerosis | | Phase 3 | | 400 | | 01/01/2003 | | Yes | | McGill | | SOM | | ND | | N/A | | N/A | | ND | | Yes | | Yes | | ND | | EC |
|  |  | |  | | PMID:19449238 | | 174 | | Recombinant human granulocyte-colony stimulating factor administration for treating amyotrophic lateral sclerosis: A pilot study. | | ND | | 39 | | ND | | Yes | | McGill | | SOM | | ND | | N/A | | N/A | | ND | | Yes | | Yes | | ND | | EC |
|  | ND | | 2018-000142-18 | |  | | 147 | | Feasibility and pharmacodynamics of subcutaneously given combination of dexmedetomidine and ketamine infusion in ALS patients receiving palliative care | | Phase 4 | | 20 | | 09/05/2018 | | Yes | | ESAS | | POM | | Yes | | ND | | EC | | ND | | Yes | | Yes | | ND | | EC |
|  | NCT02781454 | |  | |  | | 67 | | Mexiletine in Sporadic Amyotrophic Lateral Sclerosis | | Phase 2 | | 60 | | 01/10/2016 | | ND | | N/A | | N/A | | Yes | | ND | | EC | | No | | ND | | Yes | | ND | | EC |
|  | NCT01849770 | |  | | PMID:26911633 \| PMCID:PMC4836879 | | 76 | | Mexiletine in Sporadic Amyotrophic Lateral Sclerosis (SALS) | | Phase 2 | | 75 | | 01/07/2013 | | ND | | N/A | | N/A | | Yes | | ND | | EC | | No | | ND | | Yes | | ND | | EC |
|  | ND | | 2009-010060-41 | |  | | 138 | | Evaluation of efficacy of lithium salts therapy in patients with Amyotrophic Lateral Sclerosis | | Phase 2 | | 40 | | 24/02/2009 | | ND | | N/A | | N/A | | Yes | | History | | EC | | No | | ND | | Yes | | WAIS & DSM | | EC |
|  | | | | | | | | | | | | | | | | | | | | | | | | | | | | | | | | | | | | | |
| **Abbreviations** | | | | | | | | | | | | | | | | | | | | | | | | | | | | | | | | | | | | | |
| No Data Available (ND)  Not Applicable (N/A)  NCT (Clinical Trials.Gov)  Trial Identifier (Trial ID)  PubMed Identifier (PMID/PMCID)  Exclusion Criteria (EC)  Primary Outcome MEASURE (POM)  Secondary Outcome Measure (SOM)  Quality of Life (QoL)  Amyotrophic Lateral Assessment Questionnaire-40 Item (ALSAQ-40)  Amyotrophic Lateral Assessment Questionnaire-5 Item (ALSAQ-5)  ALS Specific Quality of Life (ALSQOL-R)  EuroQol 5 Domain Assessment (EQ-5D-5L)  Edmonton Symptom Assessment System (ESAS)  Schedule for the Evaluation of Individual Quality of Life Questionnaire (SEIQoL) | | | | | | | | | | | | | | | | | | | Short Form Patient Questionnaire – 12 Item (SF-12)  Short Form Patient Questionnaire – 36 Item (SF-36)  Sickness Impact Profile (SIP)  Visual Analogue Scale (VAS)  Patient’s Global Impression of Change (PGIC)  Hamilton Depression (HAM-D)  Hospital Anxiety and Depression Scale (HADS)  NeuroPsychiatric Inventory (NPI)  Columbia Suicide Severity Rating Scale (C-SSRS)  ALS-Depression-Inventory (ADI-12)  Edinburgh Cognitive and Behavioural ALS Screen (ECAS)  Montreal Cognitive Assessment (MoCA)  Frontal Behavioural Inventory (FBI)  Addenbrooke’s Cognitive Examination (ACE)  Mini Mental State Examination (MMSE)  Diagnostic Statistical Manual of Mental Disorders (DSM)  Weschler Adult Intelligence Scale (WAIS) | | | | | | | | | | | | | | | | | | |
